# Serum proteome profiles in patients treated with targeted temperature management after out-of-hospital cardiac arrest

**DOI:** 10.1186/s40635-023-00528-0

**Published:** 2023-07-17

**Authors:** Gabriele Lileikyte, Anahita Bakochi, Ashfaq Ali, Marion Moseby-Knappe, Tobias Cronberg, Hans Friberg, Gisela Lilja, Helena Levin, Filip Årman, Sven Kjellström, Josef Dankiewicz, Christian Hassager, Johan Malmström, Niklas Nielsen

**Affiliations:** 1 Department of Clinical Sciences Lund, Anaesthesia and Intensive Care, Lund University, Helsingborg Hospital, Svartbrödragränden 3, 251 87 Helsingborg, Sweden; 2 Swedish National Infrastructure for Biological Mass Spectrometry (BioMS), Lund University, Lund, Sweden; 3 Department of Clinical Sciences Lund, Infection Medicine, Lund University, Lund, Sweden; 4 National Bioinformatics Infrastructure Sweden (NBIS), SciLifeLab, Department of Immunotechnology, Lund University, Lund, Sweden; 5 Department of Clinical Sciences Lund, Neurology, Lund University, Skåne University Hospital, Lund, Sweden; 6 Department of Clinical Sciences Lund, Anaesthesia and Intensive Care, Lund University, Skåne University Hospital, Malmö, Sweden; 7 Department of Clinical Sciences Lund, Department of Research and Education, Lund University, Skåne University Hospital, Lund, Sweden; 8 Department of Clinical Sciences Lund, Cardiology, Lund University, Skåne University Hospital, Lund, Sweden; 9 Department of Cardiology, Rigshospitalet and Dept of Clinical Medicine, University of Copenhagen, Copenhagen, Denmark

**Keywords:** Out-of-hospital cardiac arrest, Heart arrest, Proteomics, Prognostication, Temperature control, Hypothermia, Targeted temperature management

## Abstract

**Background:**

Definition of temporal serum proteome profiles after out-of-hospital cardiac arrest may identify biological processes associated with severe hypoxia–ischaemia and reperfusion. It may further explore intervention effects for new mechanistic insights, identify candidate prognostic protein biomarkers and potential therapeutic targets. This pilot study aimed to investigate serum proteome profiles from unconscious patients admitted to hospital after out-of-hospital cardiac arrest according to temperature treatment and neurological outcome.

**Methods:**

Serum samples at 24, 48, and 72 h after cardiac arrest at three centres included in the Target Temperature Management after out-of-hospital cardiac arrest trial underwent data-independent acquisition mass spectrometry analysis (DIA-MS) to find changes in serum protein concentrations associated with neurological outcome at 6-month follow-up and targeted temperature management (TTM) at 33 °C as compared to 36 °C. Neurological outcome was defined according to Cerebral Performance Category (CPC) scale as “good” (CPC 1–2, good cerebral performance or moderate disability) or “poor” (CPC 3–5, severe disability, unresponsive wakefulness syndrome, or death).

**Results:**

Of 78 included patients [mean age 66 ± 12 years, 62 (80.0%) male], 37 (47.4%) were randomised to TTM at 36 °C. Six-month outcome was poor in 47 (60.3%) patients. The DIA-MS analysis identified and quantified 403 unique human proteins. Differential protein abundance testing comparing poor to good outcome showed 19 elevated proteins in patients with poor outcome (log_2_-fold change (FC) range 0.28–1.17) and 16 reduced proteins (log_2_(FC) between − 0.22 and − 0.68), involved in inflammatory/immune responses and apoptotic signalling pathways for poor outcome and proteolysis for good outcome. Analysis according to level of TTM showed a significant protein abundance difference for six proteins [five elevated proteins in TTM 36 °C (log_2_(FC) between 0.33 and 0.88), one reduced protein (log_2_(FC) − 0.6)] mainly involved in inflammatory/immune responses only at 48 h after cardiac arrest.

**Conclusions:**

Serum proteome profiling revealed an increase in inflammatory/immune responses and apoptosis in patients with poor outcome. In patients with good outcome, an increase in proteolysis was observed, whereas TTM-level only had a modest effect on the proteome profiles. Further validation of the differentially abundant proteins in response to neurological outcome is necessary to validate novel biomarker candidates that may predict prognosis after cardiac arrest.

**Supplementary Information:**

The online version contains supplementary material available at 10.1186/s40635-023-00528-0.

## Background

Adverse outcome in cardiac arrest patients who initially achieve return of spontaneous circulation (ROSC) is largely due to cerebral and cardiac dysfunction induced by the prolonged whole-body ischaemia and subsequent reperfusion injury [1, 2]. Oxygen debt, cell death, and formation of free radicals during ischaemia–reperfusion induce endothelial toxicity and a generalised activation of immunological and coagulation pathways [3–5]. Due to limited energy supplies and high metabolism, the brain is particularly vulnerable to ischaemia–reperfusion, contributing significantly to the mortality and morbidity after out-of-hospital cardiac arrest (OHCA) [2, 6].

Whole body hypothermia was introduced in post-resuscitation care when trials suggested neuroprotective effects [7, 8]. Preclinical data indicate that hypothermia exerts neuroprotection through diverse mechanisms, such as lowered cell metabolism, diminished excitotoxicity, reduced inflammation, modified gene expression, and diminished apoptosis if applied prior to, during, or early after cardiac arrest [9]. However, the Target Temperature Management after out-of-hospital cardiac arrest (TTM) trial showed no benefit of a target temperature of 33 °C as compared to 36 °C in adult patients in need of intensive care when reaching the target temperature (< 34 °C) within 4–6 h after the arrest [10]. This sparked a debate about the optimal target temperature for temperature control as well as the optimal timing for the intervention. Recent systematic reviews indicate that TTM to hypothermic temperatures *post cardiac arrest* does not confer benefit in terms of mortality and functional outcome and recent guidelines recommend only actively preventing fever [11–13]. Proteomic profiles comparing different levels of TTM could help give a mechanistic understanding of the clinical results.

Multi-modal neuroprognostication is of critical importance to guide decisions on level of intensive care after cardiac arrest, and blood biomarkers have emerged as an important component. Serial measurements of the protein neuron-specific enolase are recommended in clinical guidelines, with elevated and increasing values indicative of neuronal damage and poor prognosis [14]. The proteins neurofilament light chain, serum tau and glial fibrillary acidic protein may provide even better neuroprognostication but are not yet routinely in use [15–17]. A systematic identification and validation of other protein biomarkers may facilitate a more accurate and earlier neurological prognostication.

Continuous advances in proteomic research have established mass spectrometry (MS)-based quantitative proteomic profiling as an analytical tool in cardiovascular medicine [18]. Through application of liquid chromatography and tandem mass spectrometry (LC–MS/MS) capabilities, large numbers of proteins can be identified and quantified in detail. Findings from recent proteomic studies in OHCA patients suggest differences in proteome profiles according to both neurological outcome and temperature management [19–23]. Compared to the previous proteomics studies, we included patients from multiple centres and provide a randomised large pilot cohort with serum samples from multiple time points. The aim of this pilot study was to use the quantitative capabilities of LC–MS/MS-based proteomics to explore differences in protein abundance in relation to the temperature intervention and neurological outcome. In addition, we wanted to use proteomics to describe biological processes after OHCA and TTM, to discover possible novel biomarkers and new therapeutic targets.

## Methods

The TTM-trial prospectively included unconscious patients after OHCA with a presumed cardiac cause of arrest and randomised them to TTM at either 33 °C or 36 °C (NCT01020916). The trial design and main outcomes have been published [10, 24]. Serum samples from the three participating sites in Scania region of Sweden included in the TTM trial were used for this study. Serum samples were collected at 24, 48, and 72 h after ROSC, pre-analytically processed on site, aliquoted, and frozen to − 80 °C before shipment to the Integrated BioBank of Luxembourg as published [25]. Neurological outcome was assessed by a face-to-face follow-up visit six months after OHCA reported according to the Cerebral Performance Category (CPC) scale as “good” (CPC 1–2, good cerebral performance or moderate cerebral disability) or “poor” (CPC 3–5, severe cerebral disability, unresponsive wakefulness syndrome, or death) [10, 26, 27]. The trial was approved by the Regional Ethical Review Board in Lund, Sweden 2009/228, 2011/117. LC–MS/MS analysis was performed November 2021.

### Mass spectrometry analysis

Serum samples for de novo sequencing were prepared as described in Additional file 4: Material S1. All peptide analyses were performed on a Q Exactive HF-X Orbitrap mass spectrometer (Thermo Fisher Scientific) connected to EASY-nLC 1200 UHPLC system with a trap column (PepMap100 C18 3 µm; 75 µm × 2 cm; Thermo Fisher Scientific) and EASY-Spray column (ES803, column temperature 45 °C; Thermo Fisher Scientific). Data-independent acquisition (DIA) was performed with 44 variable windows. Solvent A was used as a stationary phase (0.1% formic acid (FA), and solvent B (mobile phase; 0.1% FA, 80% acetonitrile) was used to run a non-linear gradient from 5 to 38% over 90 min at a flow rate of 350 nl/min. Full MS scans were performed at 60,000 @ 200 m/z between mass range 350–1650 m/z.

### Data-independent acquisition and data analysis

Data-independent acquisition search was performed using *Spectronaut* version 14.10.201222.47784 on OS Windows 10 64bit. The search mode was set to library free (directDIA) using the reviewed human reference UniProt proteome database (accessed on November 2019) with isoforms with standard BGS factory settings where both precursor and protein identification q-value cutoff was set to 1%.

### Outcomes and stratifications

The main outcome for the statistical analysis was differential protein abundance at 24, 48, and 72 h after ROSC expressed as log_2_-fold changes (FC). Differentially abundant proteins were stratified according to neurological outcome (poor outcome versus good outcome) and allocation to temperature group (33 °C versus 36 °C) [10]. Generated log_2_(FC) for the differentially abundant proteins were referred to throughout the text as ‘elevated’ or ‘reduced’, indicating the direction of abundance in respective stratification comparisons.

### Statistical analyses

Demographic data were analysed using independent samples *t*-test, Pearson Chi-square test, or Mann–Whitney *U* test as appropriate, conducted using SPSS version 28.0 (SPSS, Chicago, IL). All proteomics analyses were performed using the R software: A Language and Environment for Statistical Computing [28].

Acquired data from the DIA analysis were analysed for patterns in missingness using the *mice* package to identify any samples of proteins. Proteins missing in more than 30% of the samples were filtered out to not increase the multiple testing burden and to increase the chance of plausible candidate protein replication (Additional file 4: Fig. S1). Data LOESS (locally weighted regression) normalisation was performed using the *limma* package to remove systematic effects occurring due to technical differences between assays [29].

Principal component analyses (PCA) were used to perform exploratory analyses of the proteomics data [30]. The likely differentially abundant proteins were extracted from a linear model fit using the *toptable* function. Benjamini–Hochberg and Benjamini–Yekutieli linear step-up procedures were applied to control the false discovery rate (FDR) and the expected proportion of false discoveries among the rejected hypotheses. The analyses code is available at *GiThub* repository [31].

Individual protein descriptions with their biological functions were annotated using *Uniprot* database (accessed on 2022-10-23) and their accordant studies (Additional file 4: Table S1) [32].

For production of the heat maps, significantly differentially regulated proteins (adjusted p-value $<$ 0.05) at any time point for respective predictor variable were extracted from the normalised data. Plots of respective log_2_(FC) were plotted in a heat map. Proteins with a log_2_(FC) $>$ 1 or $<$− 1 were considered of high abundance.

Biological functions of the differentially abundant proteins were analysed with *Metascape* using Gene Ontology (GO) and pathway investigation [33]. Gene Ontology refers to a controlled vocabulary composed of “GO terms” describing molecular actions, biological processes, and cellular location of gene products [34]. Analyses therefore included molecular function of gene products, biological processes in which those functions occur, and cellular component categories for the proteins acquired through the DIA-MS and statistical analyses.

Interaction analysis was performed to identify proteins associated with both neurological outcome and temperature treatment using the following formula:

$$Lo{g}_{2\left(Protein Intensity\right)}\sim Temperature Category+CPC category+ Temperature Category\times CPC category.$$

## Results

### Patients

**Fig. 1:**
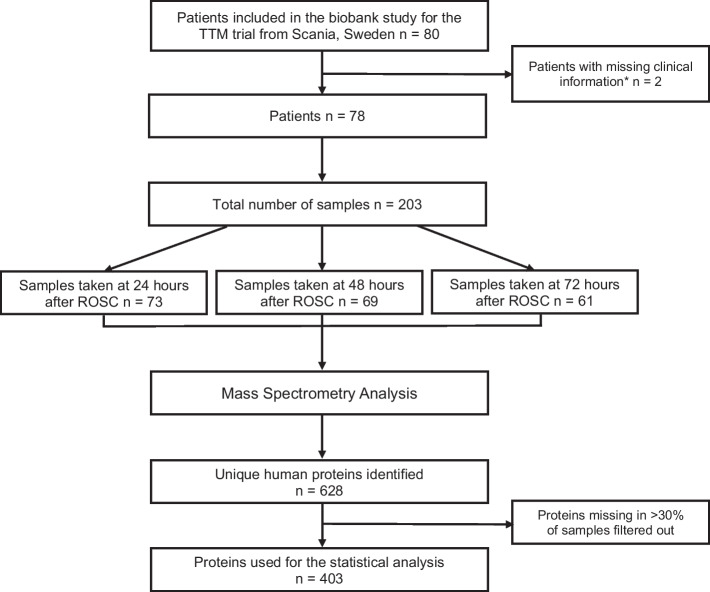
Flowchart of the patients and serum samples included in the study. *One patient was mislabeled between the mass spectrometry- and clinical data sheet; included 3 samples. A second patient was incorrectly identified between the mass spectrometry- and clinical data sheet; included 3 samples. *TTM* Target Temperature Management after out-of-hospital cardiac arrest trial

Serum samples from 78/80 eligible patients were included in the analysis (Fig. 1). Demographic data of the study population were stratified according to good outcome [31 patients (40%)] and poor neurological outcome [47 patients (60%)] evaluated at 6 months, and TTM allocation at 33 °C and 36 °C (Table 1). The two patients with missing data were allocated to the temperature treatment of 36 °C, had poor outcome, and comparable characteristics with the included patients (Additional file 4: Table S2). The number of samples available at 24, 48, and 72 h were 73, 69, and 61, respectively.

**Table 1:** Demographic characteristics of the study population stratified according to neurological outcome and temperature treatment

Characteristic^a^	Good outcome (N = 31)	Poor outcome (N = 47)	33 °C group (N = 41)	36 °C group (N = 37)
Demographic characteristics				
Age in years	62 ± 11	69 ± 12	64 ± 13	68 ± 11
Male sex	27 (87)	35 (75)	36 (88)	26 (70)
Medical history				
Chronic heart failure	1/28 (4)	4/44 (9)	1/39 (3)	4/33 (12)
Previous acute myocardial infarction	5/28 (18)	9/44 (21)	8/39 (21)	6/33 (18)
Ischaemic heart disease	5/28 (18)	12/44 (27)	9/39 (23)	8/33 (24)
Previous cardiac arrhythmia	5/28 (18)	12/44 (27)	8/39 (21)	9/33 (27)
Arterial hypertension	13/28 (46)	21/44 (48)	19/39 (49)	15/33 (46)
Previous TIA or stroke	1/28 (4)	9/44 (21)	4/39 (10)	6/33 (18)
Diabetes mellitus	1/28 (4)	9/44 (21)	3/39 (8)	7/33 (21)
Asthma or COPD	4/28 (14)	7/44 (16)	2/39 (5)	9/33 (27)
Previous percutaneous coronary intervention	2/28 (7)	5/44 (11)	4/39 (10)	3/33 (9)
Previous coronary-artery bypass grafting	0/28 (0)	5/44 (11)	5/39 (13)	0/33 (0)
Characteristics of the cardiac arrest				
Bystander witnessed cardiac arrest	28 (90)	45 (96)	37 (90)	36 (97)
Shockable rhythm	27 (87)	23 (49)	28 (68)	22 (59)
Minutes from cardiac arrest to ROSC	23 (15–33)	35 (23–50)	30 (20–43)	30 (19–48)
Clinical characteristics on admission				
First measured body temperature in °C^d^	35.9 ± 0.8	35.9 ± 0.9	35.7 ± 0.9	36.0 ± 0.8
Glasgow Coma Scale score^b,d^	5 (4–6)	3 (3–3)	3 (3–5)	3 (3–5)
Corneal reflex bilaterally present	16/26 (62)	8/44 (18)	14/38 (37)	10/32 (31)
Pupillary reflex bilaterally present	27/30 (90)	19/47 (40)	24/40 (60)	22/37 (60)
Serum pH^d^	7.3 ± 0.1	7.1 ± 0.2	7.2 ± 0.1	7.1 ± 0.2
Serum lactate in mmol/liter^d^	6.2 ± 3.7	9.2 ± 4.2	7.5 ± 4.1	8.7 ± 4.5
Circulatory shock^c^	2/28 (7)	7/44 (16)	4/39 (10)	5/33 (15)
ST-segment elevation in acute myocardial infarction	11/28 (39)	12/44 (27)	12/39 (31)	11/33 (33)
Allocation to 33 °C	20 (65)	21 (45)	41 (100)	0 (0)
Poor outcome (CPC 3–5) at 6 months	0 (0)	47 (100)	21 (51)	26 (70)

^a^Results are reported as numbers [/total number] (percentages), median (interquartile range), or mean (± standard deviation) as appropriate. *COPD* chronic obstructive pulmonary disease; *TIA* transient ischaemic attack; *ROSC* return of spontaneous circulation

^b^Scores on the Glasgow Coma Scale range from 3 to 15, with lower scores indicating reduced level of consciousness

^c^Circulatory shock was defined as a systolic blood pressure of less than 90 mm Hg for more than 30 min or end-organ hypoperfusion (cool extremities, urine output < 30 ml per hour, and a heart rate of < 60 beats per minute)

^d^Missing values. ‘First measured body temperature’ was missing data for 2 patients and ‘Glasgow Coma Scale score’ was missing data for 8 patients; ‘Serum pH’ and ‘Serum lactate’ were missing data for 6 patients

### Identification of temporal serum protein profiles

Initial data included 628 unique human proteins and 203 patient serum samples. Proteins missing in more than 30% of samples were filtered out, generating a list of 403 quantified unique human proteins used for the statistical analysis (Fig. 1). Normalised protein–patient data, detailed protein list, and clinical variable list are presented in Additional file 1: S1.

### Comparison of protein composition according to neurological outcome

**Fig. 2:**
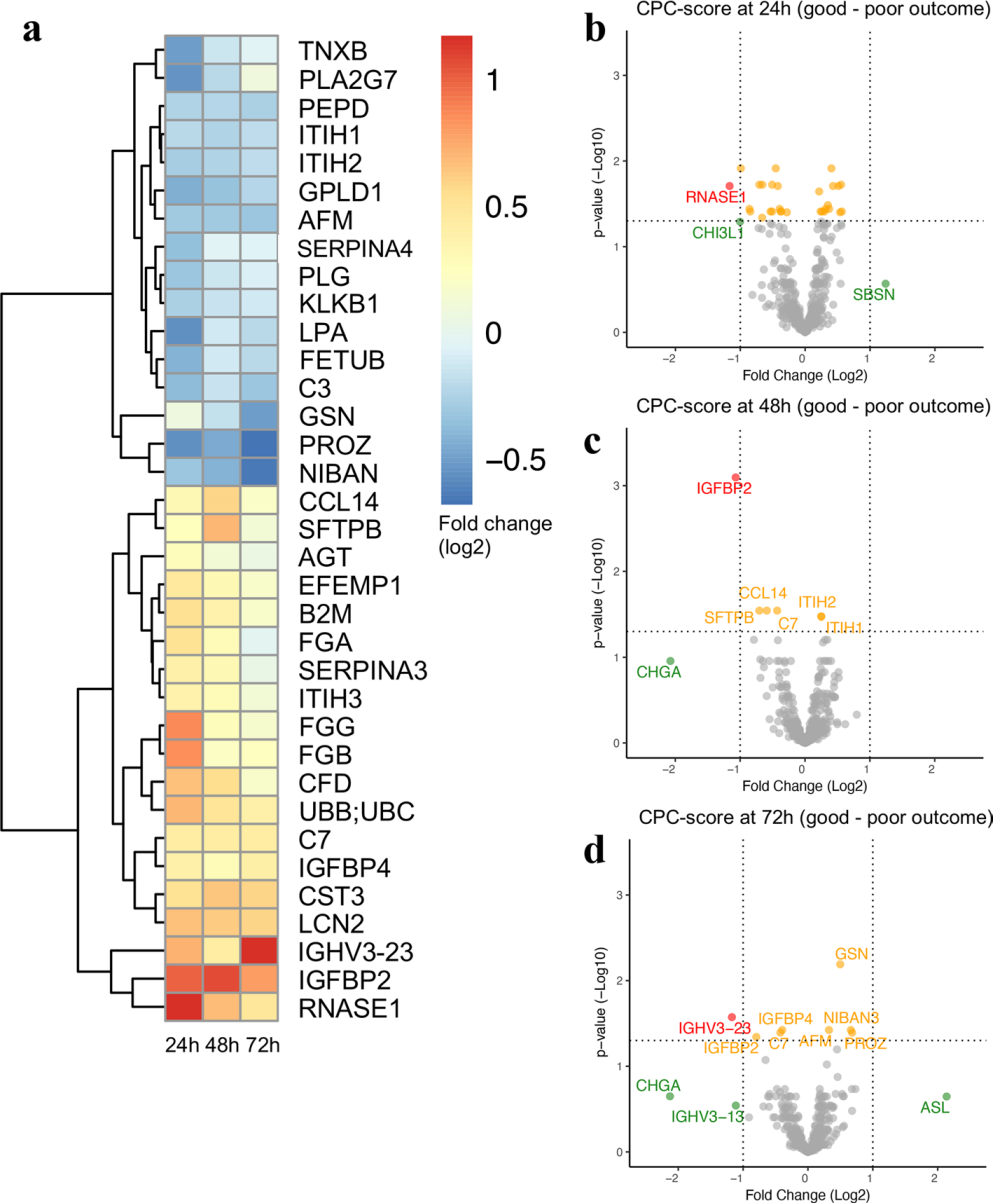
Proteomic analysis results for protein abundance according to neurological outcome. **a** Heatmap of the significantly abundant proteins at 24, 48 and 72 h after return of spontaneous circulation for poor vs. good neurological outcome. Positive log_2_-fold change (FC) indicates elevated proteins (red colour) in patients with poor outcome when compared with good outcome patients. Negative log_2_(FC) indicates reduced proteins (blue colour) in poor outcome compared with good outcome patients. **b-d** Volcano plots for the differential abundance of proteins for neurological outcome at 24 h (**b**), 48 h (**c**) and 72 h (**d**), respectively. Positive log_2_(FC) indicates good outcome, negative log_2_(FC) indicates poor outcome. Statistically significant proteins (adjusted p-value ≤ 0.05) and regulated proteins (absolute log_2_(FC) > 1) are labelled in red. Proteins with a significant adjusted p-value with a log_2_(FC) between − 1 and 1 are labelled in yellow, and statistically non-significant proteins with an absolute log_2_(FC) < − 1 or > 1 are labelled in green. Individual protein descriptions with their biological functions are presented in Additional file 4: Table S1. *AFM* afamin; *AGT* angiotensinogen; *ASL* argininosuccinate lyase; *B2M* beta-2-microglobulin; *C3* complement C3; *C7* complement component C7; *CCL14* C–C motif chemokine 14; *CFD* complement factor D; *CHGA* chromogranin-A; *CHI3L1* chitinase-3-like protein 1; *CST3* cystatin-C; *EFEMP1*-*EGF* containing fibulin-like extracellular matrix protein 1; *FETUB* fetuin-B; *FGA* fibrinogen alpha chain; *FGB* fibrinogen beta chain; *FGG* fibrinogen gamma chain; *GPLD1* phosphatidylinositol-glycan-specific phospholipase D; *GSN* gelsolin; *IGFBP2* insulin-like growth factor-binding protein 2; *IGFBP4* insulin-like growth factor-binding protein 4; *IGHV3-13* immunoglobulin heavy variable 3-13; *IGHV3-23* immunoglobulin heavy variable 3-23; *ITIH1* inter-alpha-trypsin inhibitor heavy chain H1; *ITIH2* inter-alpha-trypsin inhibitor heavy chain H2; *ITIH3* inter-alpha-trypsin inhibitor heavy chain H3; *KLKB1* plasma kallikrein; *LCN2* neutrophil gelatinase-associated lipocalin; *LPA* apolipoprotein (a); *NIBAN* protein Niban 3; *PEPD* Xaa-Pro dipeptidase; *PLA2G7* platelet-activating factor acetylhydrolase; *PLG* plasminogen; *PROS* vitamin K-dependent protein S; *RNASE1* ribonuclease pancreatic; *SBSN* suprabasin; *SERPINA3* alpha-1-antichymotrypsin; *SERPINA4* kallistatin; *SFTPB* pulmonary surfactant-associated protein B; *TNXB* tenascin-X; *UBB/UBC* polyubiquitin-B/polyubiquitin-C

Neurological outcome was associated with 29 unique statistically changed proteins at 24 h, six proteins at 48 h, and eight proteins at 72 h after ROSC (Fig. 2a, Additional file 2: S2). Serum abundances were elevated for 19 proteins (log_2_(FC) range 0.28–1.17) and reduced for 16 proteins (log_2_(FC) range between − 0.22 and − 0.68) when comparing poor outcome to good outcome. The statistical significance and log_2_(FC) value is presented in the volcano plots in Fig. 2b–d. Three significantly elevated proteins in patients with poor outcome with a log_2_(FC) $<$− 1 were noted; ribonuclease pancreatic (RNASE1) at 24 h, insulin-like growth factor-binding protein 2 (IGFBP2) at 48 h, and immunoglobulin heavy variable 3–23 (IGHV3-23) at 72 h.

Among the statistically enhanced proteins in patients with poor outcome, complement component 7 (C7) and IGFBP2 were present at all three time points. Insulin-like growth factor-binding protein 4 (IGFBP4), a regulator of insulin growth factor, was present at 24 and 72 h. None of the reduced proteins were statistically significant at all three time points but inter-alpha-trypsin inhibitor heavy chain H1 (ITIH1) was significantly reduced at both 24 and 48 h. In addition, vitamin K-dependent protein Z (PROZ) and afamin (AFM) were mutually reduced at 24 and 72 h.

**Fig. 3:**
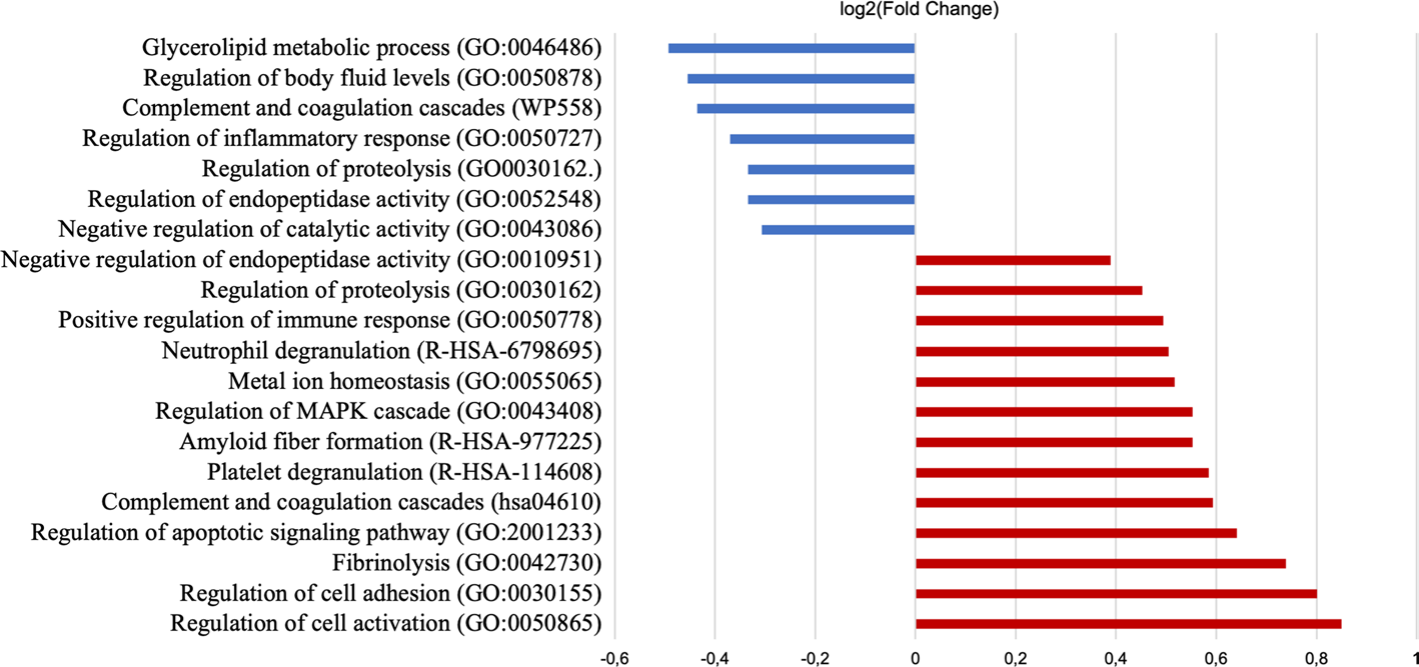
Biological processes for the differentially abundant proteins associated with neurological outcome. Proteins enhanced for neurological outcome were annotated by selected gene ontology terms for biological process. The average log_2_-fold change for all proteins included in each term is plotted to show the direction of average change for poor outcome (in red) as compared to good outcome (in blue)

Among the Gene Ontology (GO) terms for biological processes enriched for the elevated proteins in patients with poor outcome, the terms ‘positive regulation of immune response’, ‘amyloid fibre formation, ‘metal ion homeostasis’, and ‘regulation of apoptotic signalling pathway’ were notable (Fig. 3). Elevated proteins are physiologically expressed in various tissues, such as endocrine gland, occipital lobe, bone marrow, liver, pancreas, and the hematopoietic system. According to the cellular component, most of the elevated proteins can be physiologically found in endomembrane system, vesicles, secretory granules, and as blood microparticles. For the reduced proteins in patients with poor outcome, notable GO terms were ‘glycerolipid metabolic process’, ‘complement and coagulation cascades’, ‘regulation of inflammatory response’, and ‘regulation of proteolysis’ (Fig. 3). Molecular functions of the reduced proteins include endopeptidase inhibitor activity, serine-type endopeptidase activity, and apolipoprotein binding. According to cellular component, proteins reduced in poor outcome are physiologically secreted in extracellular exosomes, secretory granule lumen, and as blood microparticles. In addition, analysis of tissue expression revealed bone marrow, liver, and endocrine glands as potential tissues. The full list of enriched biological processes according to neurological outcome is presented in Additional file 4: Table S3a, b.

### Comparison of protein composition according to level of TTM

**Fig. 4:**
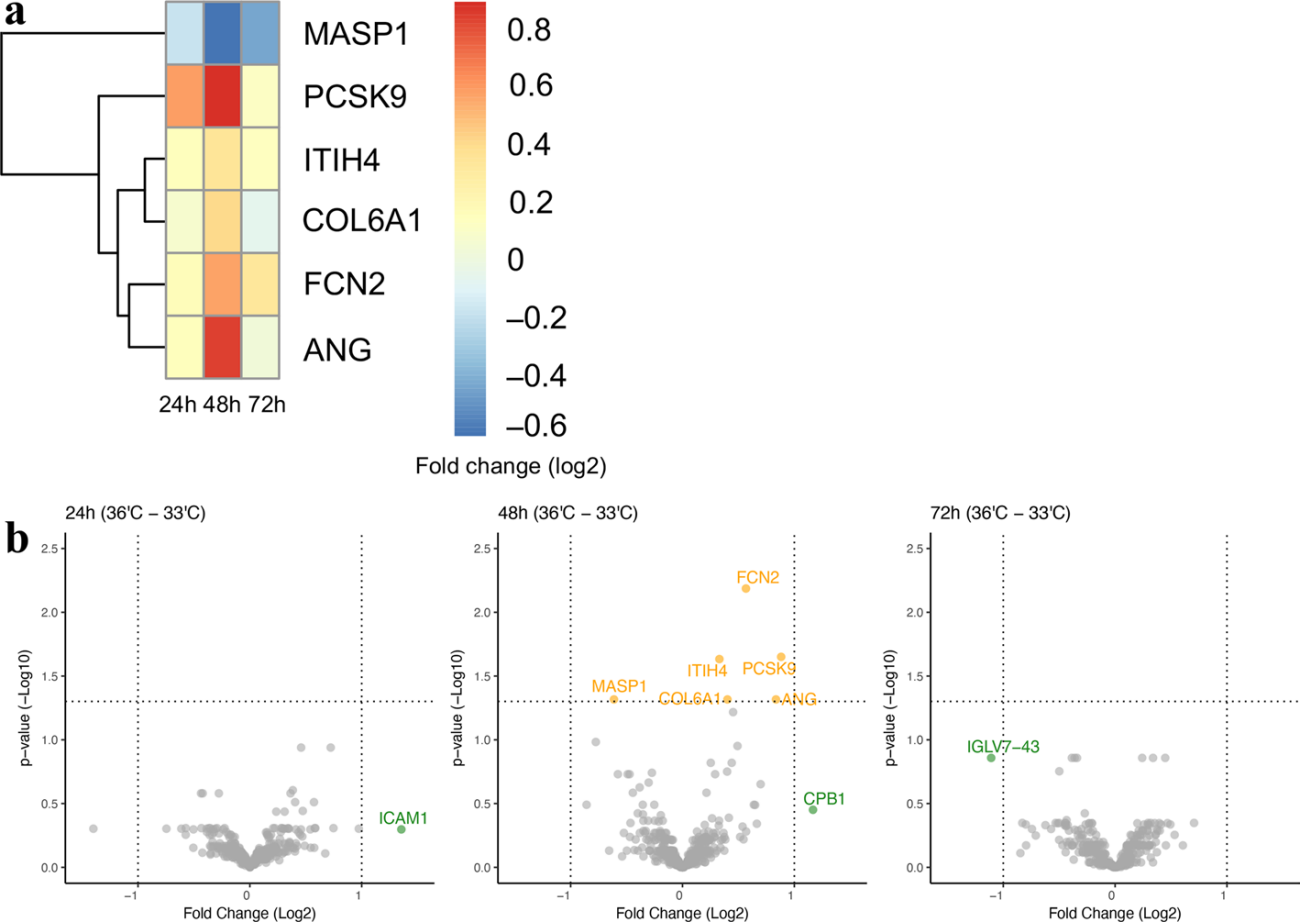
Proteomic analysis results for protein abundance according to temperature treatment of 36 °C to 33 °C. **a** Heatmap of the significantly differentially abundant proteins for comparison of temperature treatment of 36 °C to 33 °C at 24, 48 and 72 h after return of spontaneous circulation. Positive log_2_-fold change (FC) indicates elevated proteins (red colour) in patients treated at target temperature 36 °C compared to 33 °C. Negative log_2_(FC) indicates proteins reduced (blue colour) in patients treated at target temperature 36 °C versus 33 °C. **b** Volcano plots for the differential abundance of proteins between temperature treatment at 24, 48, and 72 h. Positive log_2_(FC) indicates treatment with 36 °C, negative log_2_(FC) indicates treatment with 33 °C. Proteins with a significant adjusted p-value, and with a log_2_(FC) between − 1 and 1 are labelled in yellow, and statistically non-significant proteins with an absolute log_2_(FC) <  − 1 or > 1 are labelled in green. Individual protein descriptions with their biological functions are presented in Additional file 4: Table S1. *ANG* angiogenin; *COL6A* collagen alpha-1(VI) chain; *CPB1* carboxypeptidase B; *FCN2* Ficolin-2; *ICAM1* intercellular adhesion molecule 1; *IGLV7-43* immunoglobulin lambda variable 7–43; *ITIH4* inter-alpha-trypsin inhibitor heavy chain family member 4; *MASP1* isoform 2 of mannan-binding lectin serine protease 1; *PCSK9* proprotein convertase subtilisin/kexin type 9

Comparison of the temperature groups 33° and 36 °C yielded significantly altered protein levels for six proteins at 48 h, with no significantly different protein levels at 24 or 72 h (Fig. 4a, Additional file 3: S3). When comparing the 36 °C group to the 33 °C group, serum concentrations for five proteins were elevated (log_2_(FC) range 0.33–0.88) and one was reduced (log_2_(FC) − 0.6) as presented in the volcano plots in Fig. 4b. The five enhanced proteins in the 36 °C group were angiogenin (ANG), proprotein convertase subtilisin/kexin type 9 (PCSK9), inter-alpha-trypsin inhibitor heavy chain family member 4 (ITIH4), ficolin-2 (FCN2), and collagen alpha-1(VI) chain (COL6A1). Among the GO terms for the proteins elevated in the 36 °C group, terms ‘humoral immune response, ‘opsonisation’, and ‘cholesterol homeostasis’ were notable. These proteins can be physiologically found in the extracellular space and are predominantly expressed in the liver with a few exceptions; ANG has previously been detected in spinal cord neurons, and PCSK9 is also expressed in Schwann cells and found in cerebrospinal fluid [35, 36]. The reduced protein in the 36 °C group was mannan-binding lectin serine protease 1 (MASP1), a plasma protein primarily secreted by the liver, involved in ‘cell surface pattern recognition receptor signalling pathway’ and ‘complement activation via lectin pathway’ [37]. MASP1 is physiologically located in the extracellular space and its main molecular function is peptidase activity [38].

### Cross-comparison between neurological outcome and level of TTM

**Fig. 5:**
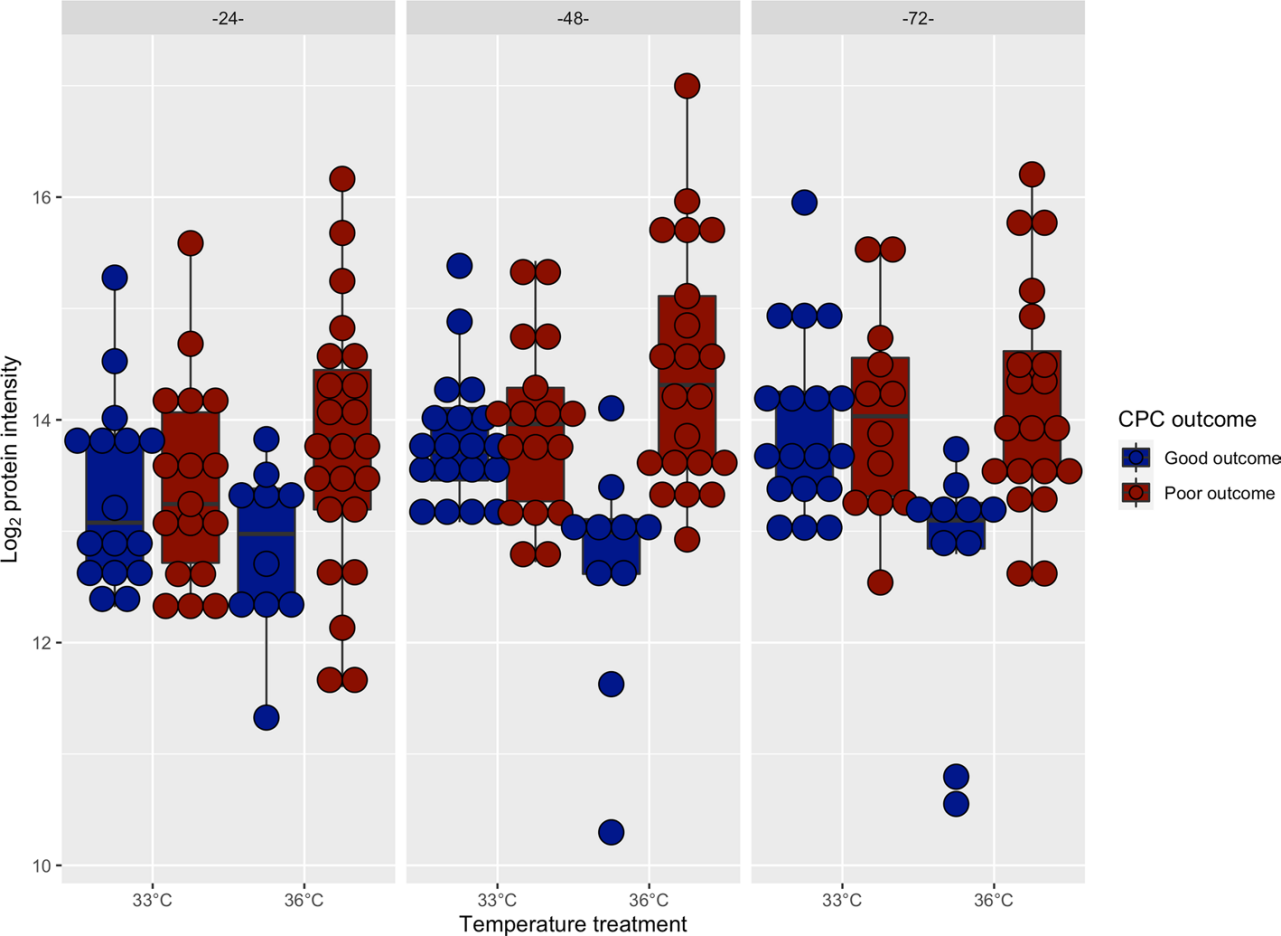
Interaction plot for Extracellular superoxide dismutase (EC-SOD) levels according to neurological outcome and temperature treatment. Results are displayed for 24, 48, and 72 h after return of spontaneous circulation. EC-SOD levels were significantly increased in poor outcome patients treated with a targeted temperature of 36 °C at 48 h compared to patients with good outcome, indicating an antioxidative response. No statistically significant differences could be seen between good and poor outcome in patients treated with targeted temperature of 33 °C. *CPC* Cerebral Performance Category

Interaction analysis between neurological outcome and temperature treatment detected only significant differences in abundance of extracellular superoxide dismutase (EC-SOD). EC-SOD, a predominantly extracellular antioxidant enzyme, was enhanced in patients with poor outcome in the 36 °C group as compared to patients with good outcome in the 36 °C group (*p*_*interaction*_ < 0.001, adjusted *p* = 0.17 for the 48-h time point, Fig. 5) [39]. No significant differences in EC-SOD regulation according to neurological outcome could be seen in patients in the 33 °C group.

## Discussion

This pilot study analysed changes in proteomic profiles of OHCA-patients according to neurological outcome and targeted temperature management (TTM). Comparison of poor and good outcome yielded differences in 35 proteins at 24, 48, and 72 h after cardiac arrest, while TTM at 33 °C or 36 °C was associated with statistically significant changes in regulation of six proteins only at 48 h. Interaction analysis between neurological outcome and level of TTM showed a significant association for higher levels of the antioxidant EC-SOD in poor outcome patients in the 36 °C group.

Proteomics represents the large-scale analysis of proteomes that can be used for explorative and unbiased discovery of temporal serum proteome profile differences between study groups. Such analysis has the potential to discover novel biomarkers, as the unbiased nature of the analysis assists identification of proteins that might otherwise not have been considered relevant in the clinical situation. Identified candidate proteins should then be tested prospectively. In this study, comparison of proteomic profiles between patients with poor and good outcome demonstrated proteins involved in e.g., inflammatory/immune responses, apoptosis, metal ion homeostasis, and proteolysis. Many of the significantly abundant proteins were enhanced in patients with poor outcome, suggesting a lower abundance of proteins associated with, for example, apoptosis in patients with good outcome. In comparison to previous proteomic studies in OHCA patients, our study reveals contrasting proteomic profiles according to neurological outcome [19–22].

Previously described neurological biomarkers for cardiac arrest, such as neuron-specific enolase, neurofilament light chain, and total-tau were not detected in the current analysis, most likely due to their low concentration in serum, which falls below the detection limit of the MS-analysis [40]. As serum albumin and immunoglobulins account for a large proportion of serum protein concentration, this can prevent detection and identification of less abundant proteins in serum [41]. Conversely, depletion of serum albumin from samples can lead to subsequent removal of low-abundant biomarkers that may bind to albumin as carrier protein [40, 42]. By using fractionation techniques combined with intermittent depletion of high-abundant proteins followed by targeted MS analysis such as parallel reaction monitoring MS, the neurological brain injury markers might be identified in future studies [43].

The prognostic biomarkers studied in patients after cardiac arrest focus on neurological outcome prediction due to the high mortality risk following hypoxic brain injury [6, 44]. However, the substantial mortality in resuscitated patients after OHCA can also depend on, e.g., the post-ischaemic cardiac dysfunction [1]. Although CPC score has been defined as a functional outcome highly associated with severe brain injury, other factors may have importance for the outcome, such as frailty prior to arrest, death due to a non-neurological cause, presumed wishes of patients, or regional differences in rating quality of life with sustained disabilities [45]. It is therefore of interest to analyse broader serum proteomic changes in combination with clinical variables in OHCA patients to better define the distinct phenotypes of the cardiac arrest population and reasons for death after resuscitation. We identified several proteins of interest as potential biomarkers, such as IGFBP2, PROZ, NIBAN, kallistatin and angiotensinogen [21]. Angiotensinogen, a component of the renin–angiotensin system (RAS), is mainly produced by astrocytes and at low levels in neurons within the brain [46–48]. As cardiac arrest-induced brain injury causes disruption in the blood–brain barrier, leakage of brain-specific proteins can be measured in serum and plasma samples [3]. Hyperactivation of RAS in the nigrostriatal system has been proposed to exacerbate oxidative stress and microglial inflammatory response, which could subsequently be inhibited by RAS-inhibitors [48]. The proteins identified in our study should be validated in larger and prospectively collected materials before any candidate prognostic markers could be suggested.

Comparison of proteomic profiles between patients treated with a target temperature of 33 °C and 36 °C revealed a small fraction of proteins involved in inflammatory/immune responses, vascularisation, cholesterol homeostasis, and neuronal apoptotic processes [49–51]. According to the TTM-trial, temperature intervention and rewarming to normothermia concluded at 36 h after ROSC, while changes in protein abundance in this pilot study were only significant for the 48-h time point, indicating no significant changes during temperature intervention at the 24-h time point [10, 24]. The majority of proteins were enhanced in the 36 °C group, suggesting a decrease in e.g., local inflammatory response in the 33 °C group after rewarming. Previous analyses of serum samples from the TTM-trial have demonstrated that TTM at 33 °C compared with 36 °C does not significantly influence specific mediators of inflammatory response, such as interleukins, tumour necrosis factor-α, C-reactive protein, and procalcitonin [52, 53]. None of these mediators were significantly altered in our pilot study.

We found decreased levels of PCSK9 in the 33 °C group compared to the normothermia group. As PCSK9 is a regulator of neuronal apoptosis and its inhibition in rat models was associated with reduced brain inflammation in cardiac ischaemia–reperfusion injury, the decreased levels of PCSK9 could suggest a theoretical neuroprotective pathway in hypothermia [54–56]. Although hypothermia has been previously shown in animal models to have a role in neuroprotection, large recent trials in humans show no difference in long-term neurological outcome as opposed to normothermia [57–59]. Furthermore, the low number of differentially regulated proteins between the temperature groups (total number of regulated proteins *N* = 6), suggests minimal changes during and after temperature intervention, supporting the clinical conclusion of the TTM-trial that showed no significant differences in mortality or neurological outcome [10].

EC-SOD is an antioxidant enzyme that neutralises reactive oxygen species from detrimental effects of systemic ischaemia–reperfusion response in post-cardiac arrest syndrome [3, 39, 60].

EC-SOD is not brain-specific, but within the brain, EC-SOD is predominantly localised in neurons in the hippocampus, thalamus and hypothalamus and is expressed in response to hypoxia [61, 62]. We found enhanced EC-SOD regulation in poor outcome patients treated at 36 °C but not at 33 °C, possibly suggesting an active protection process against hypoxia in the higher temperature group [63, 64]. Previous studies indicate contrasting results, while animal studies found decreased levels of free radicals in hypothermia, OHCA patients treated with mild hypothermia showed significantly decreased cytosolic/mitochondrial SOD enzyme activity and a significant increase in reactive oxygen species compared to healthy volunteers [60, 65, 66].

### Strengths and limitations

To our knowledge, this is the largest sample cohort used to date for a quantitative LC–MS/MS-analysis in OHCA patients and the first with samples from a randomised trial. Prospectively collected serial measurements allowed sequential analyses of changes in protein abundance over time. The structured follow-up at six months and a conservative approach to neurological prognostication is a strength of the TTM-trial [10]. Data analysis was performed using DIA, allowing less stochastic analysis compared to data-dependent acquisition (DDA). Furthermore, the generated DIA files can be retrospectively analysed against another assay library for further exploration.

TTM was initiated within two hours after OHCA and thus this study cannot exclude that protein abundance according to level of TTM and the associated biological functions could be altered by initiating induction of hypothermia at an earlier time point [10]. As systematic serum sampling was applied during the initial trial, we cannot exclude that this sampling method is not necessarily reflective of brain-specific pathology as compared to cerebrospinal fluid [10]. Usage of serum instead of plasma samples may have altered or limited the number of proteins discovered by DIA-MS analysis, although previous studies suggest better reproducibility of serum samples by MS-analysis [67]. Sample collection for the TTM-trial was performed a decade ago, which could have affected quantity, quality, and reactivity in protein samples [68]. Serum samples might be affected by repeated freeze–thaw cycles, resulting in degradation of proteins and creation of insoluble precipitates, whereas confirmation of previous measurements could be indicated [69]. Additionally, the freezing process of serum can cause precipitation and possible denaturation of proteins and we cannot exclude that this may have had an influence on our results.

Limitations further include that sample preparation and the sub sequential MS-analysis were performed in separate batches at different time points, resulting in batch effect as sample preparation was performed manually. By facilitating liquid handlers for performing sample preparation steps this error may be removed in the future studies. Proteome profiles might have been affected by non-cerebral causes of death, such as multi-organ failure and haemodynamic failure, thus substantial confounding cannot be excluded in the poor vs. good outcome groups. We have not examined the influence of any other possible confounders on our results. As proteins missing in more than 30% of the samples were filtered out, biologically interesting proteins were potentially removed, which could be avoided in future larger studies by restricting protein filtration to a later stage of data analysis. Reproducibility of proteomic analysis in biological samples is challenging due to varying sensitivity of applied methods and data-dependent statistical sampling, warranting validation studies using replication in an independent cohort or different methods [70].

## Conclusions

Liquid chromatography and tandem mass spectrometry identified protein profiles associated with neurological outcome. Poor outcome patients demonstrated increased responses in inflammatory, immunity regulating, and apoptotic proteins, whilst good outcome patients more often had increased proteolysis. Temperature management had little effect on protein abundance. Further validation is necessary in search for novel biomarkers that may correlate with prognosis after cardiac arrest.

## Supplementary Information

**Additional file 1: S1a.** The full protein–patient list after Loess normalisation. **S1b.** Protein list of the unique human proteins detected and quantified through DIA-MS. **S1c.** Clinical variables for the patients included in the pilot study.

**Additional file 2: S2a.** Protein abundances according to neurological outcome for the 24-hour time point. **S2b.** Protein abundances according to neurological outcome for the 48-hour time point. **S2c.** Protein abundances according to neurological outcome for the 72-hour time point.

**Additional file 3: S3a.** Protein abundances according to level of TTM for the 24-hour time point. **S3b.** Protein abundances according to level of TTM for the 48-hour time point. **S3c.** Protein abundances according to level of TTM for the 72-hour time point.

**Additional file 4: Material S1.** Supplemental methods, Sample preparation for de novo sequencing. **Table S1.** Specific protein descriptions for the differentially abundant proteins according to neurologic outcome and temperature treatment. **Table S2. **Demographic characteristics of the whole study population stratified according to included and excluded patients. **Table S3a. **Full list of enriched biological processes for the elevated proteins according to neurologic outcome. **Table S3b. **Full list of enriched biological processes for the reduced proteins according to neurologic outcome. **Figure S1. **QC plots for the missing protein values in the proteomics data.

## Data Availability

The mass spectrometry proteomics data generated and analysed during the current study have been deposited to the ProteomeXchange Consortium via the PRIDE partner repository with the dataset identifier PXD040592.

## Abbreviations

AFM: Afamin
ANG: Angiogenin
ApoER2: Apolipoprotein E receptor 2
C7: Complement component 7
CHGA: Chromogranin A
COL6A1: Collagen alpha 1 (VI) chain
CPC: Cerebral performance category
DDA: Data-dependent acquisition; shotgun mass spectrometry
DIA: Data-independent acquisition
EC-SOD: Extracellular superoxide dismutase
FA: Formic acid
FC: Fold change
FCN2: Ficolin-2
FDR: False discovery rate
GO: Gene Ontology
IGFBP2: Insulin-like growth factor binding protein 2
IGFBP4: Insulin-like growth factor-binding protein 4
IGHV3-23: Immunoglobulin heavy variable 3-23
IL: Interleukin
ITIH1: Inter-alpha-trypsin inhibitor heavy chain H1
ITIH4: Inter-alpha-trypsin inhibitor heavy chain family member 4
LC–MS/MS: Liquid chromatography and tandem mass spectrometry
LOESS: Locally weighted regression
MASP1: Mannan-binding lectin serine protease 1
MS: Mass spectrometry
NF-kB: Nuclear factor kappa-light-chain-enhancer of activated B cells
OHCA: Out-of-hospital cardiac arrest
PCA: Principal component analyses
PCSK9: Proprotein convertase subtilisin/kexin type 9
PROZ: Vitamin K-dependent protein Z
RAS: Renin–angiotensin system
RNASE1: Ribonuclease pancreatic
ROSC: Return of spontaneous circulation
TTM: Targeted temperature management
