# Supplementary material for: Serum proteome profiles in patients treated with targeted temperature management after out-of-hospital cardiac arrest
Source: Intensive Care Med Exp. 2023 Jul 17;11:43. doi: 10.1186/s40635-023-00528-0 (PMC10350448; doi:10.1186/s40635-023-00528-0)
Supplement: Supplementary file 4 — Additional file 4: Material S1. Supplemental methods, Sample preparation for de novo sequencing. Table S1. Specific protein descriptions for the differentially abundant proteins according to neurologic outcome and temperature treatment. Table S2. Demographic characteristics of the whole study population stratified according to included and excluded patients. Table S3a. Full list of enriched biological processes for the elevated proteins according to neurologic outcome. Table S3b. Full list of enriched biological processes for the reduced proteins according to neurologic outcome. Figure S1. QC plots for the missing protein values in the proteomics data. [file 40635_2023_528_MOESM4_ESM.docx]

# Additional file for *Serum proteome profiles in patients treated with targeted temperature management after out-of-hospital cardiac arrest*

Gabriele Lileikyte, Anahita Bakochi, Ashfaq Ali, Marion Moseby-Knappe, Tobias Cronberg, Hans Friberg, Gisela Lilja, Helena Levin, Filip Årman, Sven Kjellström, Josef Dankiewicz, Christian Hassager, Johan Malmström, Niklas Nielsen

## Additional file methods S1

## Sample preparation for de novo sequencing

Serum samples (5 microliter (µl)) were incubated for 30 minutes at 37 °C in 8M urea and 10mM dithiothreitol for denaturation and reduction. The samples were then alkylated by 20 mM 2-iodoacetamide for 60 minutes at room temperature in dark. Afterwards, 25 µl was transferred into a new vial, and digested with 1 µg Lys-C-protease for 5 hours at room temperature. The samples were diluted with 50 mM ammonium bicarbonate in 8 M urea, and digested with 1 µg trypsin (sequence-grade modified porcine trypsin, Promega) overnight at 37 °C. The digestion was stopped by 20 µl 10 % trifluoroacetic acid (TFA). Peptide clean-up and concentration was done by using C18 columns according to manufacturer’s instructions (Nest Group, HEM S18V). The samples were resuspended in 25 µl 2 % acetonitrile, 0.1 % TFA with iRT 1:10.

**Table S1.** Specific protein descriptions for the differentially abundant proteins according to neurologic outcome and temperature treatment. UniProt accessed on 2022-10-16.

| **Gene name** | **Protein name** | **Biological function** |
| --- | --- | --- |
| ***Neurologic outcome*** | | |
| AFM | Afamin | Carrier for hydrophobic molecules in body fluids. Binds to Vitamin E (1). |
| AGT | Angiotensinogen | Component of the renin-angiotensin system. Regulator of blood pressure, fluid- and electrolyte homeostasis (2). |
| ASL | Argininosuccinate lyase | Catalyses cleavage to fumarate and L-arginine, provides hepatic nitrogen detoxification into excretable urea (3). |
| B2M | Beta-2-microglobulin | Class I major histocompatibility complex component. Involved in the presentation of peptide agents to the immune system (4). |
| C3 | Complement C3 | Plays a central role in complement system activation in both classical and alternative complement pathways (5). |
| C7 | Complement C7 | Constituent in the membrane attack complex (MAC), involved in the innate and adaptive immune response (6). |
| CCL14 | C-C motif chemokine 14 | Processed form is a chemotactic factor that attracts monocytes, eosinophils, and T-cells (7). |
| CFD | Complement factor D | Cleaves factor B from C3b complex, activating C3 convertase of the alternate pathway (8). |
| CHGA | Chromogranin A | Glycoprotein precursor to vasostatin, pancreastatin, serpinin, etc (9). |
| CHI3L1 | Chitinase-3-like protein 1 | Involved in T-helper cell inflammatory response and IL-13-induced inflammation. Regulates hyperoxia-induced injury and inflammation in lung (10). |
| CST3 | Cystatin-C | Inhibitor of cysteine proteinases that are in turn responsible for e.g., apoptosis (11). |
| EFEMP1 | EGF-containing fibulin-like extracellular matrix protein 1 | Binds epidermal growth factor receptor, activating downstream signaling pathways (12). |
| FETUB | Fetuin-B | Protease inhibitor required for egg fertilization (13). |
| FGA | Fibrinogen alpha chain | Monomer that is polymerized to fibrin, one of the primary components of blood clots (14). |
| FGB | Fibrinogen beta chain | *Same as above* |
| FGG | Fibrinogen gamma chain | *Same as above* |
| GPLD1 | Phosphatidylinositol-glycan-specific phospholipase D | Hydrolises inositol-phosphate linkage in proteins anchored by GPI-anchor, thus releasing proteins from the membrane (15). |
| GSN | Gelsolin | Calcium-regulated, actin-modulating protein that prevents monomer exchange (16). |
| IGFBP2 | Insulin-like growth factor (IGF)-binding protein 2 | Inhibits IGF-mediated growth and developmental rates (17). |
| IGFBP4 | Insulin-like growth factor (IGF)-binding protein 4 | Prolongs the half-life of IGF, alters IGFs interaction with cell-surface receptors (18). |
| IGHV3-13 | Immunoglobulin heavy variable 3-13 | V region of the variable domain of immunoglobulin heavy chains that participates in antigen recognition (19). |
| IGHV3-23 | Immunoglobulin heavy variable 3-23 | *Same as above* |
| ITIH1 | Inter-alpha-trypsin inhibitor heavy chain H1 | Carrier of hyaluronan in serum, binding protein between hyaluronan and other matrix proteins (20). |
| ITIH2 | Inter-alpha-trypsin inhibitor heavy chain H2 | *Same as above* |
| ITIH3 | Inter-alpha-trypsin inhibitor heavy chain H3 | *Same as above* |
| KLKB1 | Plasma kallikrein | Activates factor XII; converts prorenin to renin; releases bradykinin from HMW kininogen (21, 22). |
| LCN2 | Neutrophil gelatinase-associated lipocalin | Iron-trafficking protein involved in apoptosis, innate immunity, and renal development (23-25). |
| LPA | Apolipoprotein(a) | Inhibits tissue-type plasminogen activator I; promotes thrombogenesis through attachment to atherosclerotic lesions (26). |
| NIBAN3 | Protein Niban 3 | Apoptosis regulator; enriched in B-cells (27). |
| PEPD | Xaa-Pro dipeptidase | Involved in collagen metabolism due to high levels of iminoacids in collagen (28). |
| PLA2G7 | Platelet-activating factor acetylhydrolase | Part of HDL-particles, involved in phospholipid catabolism during inflammation and oxidative stress (29). |
| PLG | Plasminogen | Precursor to plasmin which degrades fibrin in blood clots; involved in ARDS (30). |
| PROZ | Vitamin K-dependent protein Z | Assists haemostasis by binding thrombin and promoting its association with phospholipid vesicles; inhibits factor Xa (31). |
| RNASE1 | Ribonuclease pancreatic | Catalyses cleavage of RNA; more commonly degrades ds-RNA (32). |
| SBSN | Suprabasin | Enriched in basal ganglia, cerebral cortex. Expressed in keratinocytes (33). |
| SERPINA3 | Alpha-1-antichymotrypsin | Unclear physiological function; can inhibit conversion to Angiotensin-2. |
| SERPINA4 | Kallistatin | Inhibits amidolytic and kininogenase activities of tissue kallikrein (34). |
| SFTPB | Pulmonary surfactant-associated protein B | Promotes alveolar stability by lowering the surface tension at the air-liquid interface in the peripheral air spaces (35). |
| TNXB | Tenascin-X | Substrate-adhesion molecule that inhibits cell migration; mediates interaction between cells and extracellular matrix (36). |
| UBB | Polyubiquitin-B | Involved in targeting of proteins for degradation, stress response, regulation of gene expression. Accumulates in neurofibrillary tangles and neuritic plaques in Alzheimers disease (37, 38). |
| ***Temperature treatment*** | | |
| ANG | Angiogenin | Stimulates ribosomal RNA-synthesis; induces vascularisation of normal and malignant tissues (39, 40). |
| COL6A1 | Collagen alpha-1(VI) chain | Cell-binding protein; maintains integrity of various tissues (41). |
| CPB1 | Carboxypeptidase B | Tissue specific for pancreas; involved in digestion (42). |
| FCN2 | Ficolin-2 | May function in innate immunity through activation of the lectin complement pathway (43, 44). |
| ICAM1 | Intercellular adhesion molecule 1 | Ligand for the leukocyte adhesion protein, binds to integrins CD11/CD18 (45, 46). |
| IGLV7-43 | Immunoglobulin lambda variable 7-43 | V-region of the variable domain of immunoglobulin light chains that participates in the antigen recognition (19). |
| ITIH4 | Inter-alpha-trypsin inhibitor heavy chain family member 4 | Type II acute-phase protein involved in inflammatory responses to trauma. May play a role in liver regeneration (47). |
| MASP1 | Isoform 2 of Mannan-binding lectin serine protease 1 | Functions in the lectin complement pathway; may be involved in coagulation (48, 49). |
| PCSK9 | Proprotein convertase subtilisin/kexin type 9 | Regulator of plasma cholesterol homeostasis and neuronal apoptosis (50, 51). |

**Table S2.** Demographic characteristics of the whole study population stratified according to included and excluded patients. *

| **Characteristic** | **Included patients**  **(N = 78)** | **Excluded patients**  **(N = 2)** | **P-value** | |
| --- | --- | --- | --- | --- |
| **Demographic characteristics** | | |  | |
| Age in years | 66±12 | 74±1 | 0.267 | |
| Male sex | 62 (80) | 2 (100) | 0.474 | |
| **Medical history** | | |  | |
| Chronic heart failure | 5/72 (7) | N/A |  | |
| Previous acute myocardial infarction | 14/72 (19) | N/A |  | |
| Ischemic heart disease | 17/72 (24) | N/A |  | |
| Previous cardiac arrhythmia | 17/72 (24) | N/A |  | |
| Arterial hypertension | 34/72 (47) | N/A |  | |
| Previous TIA or stroke | 10/72 (14) | N/A |  | |
| Diabetes mellitus | 10/72 (14) | N/A |  | |
| Asthma or COPD | 11/72 (15) | N/A |  | |
| Previous percutaneous coronary intervention | 7/72 (10) | N/A |  | |
| Previous coronary-artery bypass grafting | 5/72 (7) | N/A |  | |
| **Characteristics of the cardiac arrest** | | |  | |
| Bystander witnessed cardiac arrest | 73 (94) | 2 (100) | 0.712 |  |
| Bystander witnessed cardiac arrest | 67/72 (93) | 1/1 (100) | 0.785 | |
| Shockable rhythm | 50 (64) | 2 (100) | 0.293 | |
| Minutes from cardiac arrest to ROSC | 30 (20-44) | 21 (16-25) | 0.302 | |
| **Clinical characteristics on admission** | | |  | |
| First measured body temperature in °C ¶ | 35.9±0.8 | 35.6±0.6 | 0.587 | |
| Glasgow Coma Scale score ‡ ¶ | 3 (3-5) | N/A |  | |
| Corneal reflex bilaterally present | 24/70 (34) | 1/2 (50) | 0.645 | |
| Pupillary reflex bilaterally present | 46/77 (60) | 1/2 (50) | 0.782 | |
| Serum pH ¶ | 7.2±0.2 | N/A |  | |
| Serum lactate in mmol/liter ¶ | 8.0±4.3 | N/A |  | |
| Circulatory shock § | 9/72 (13) | N/A |  | |
| ST-segment elevation in acute myocardial infarction | 23/72 (32) | N/A |  | |
| **Allocation to 33°C** | 41 (53) | 0 (0) | 0.142 | |
| **Poor outcome (CPC 3-5) at 6 months** | 47 (60) | 2 (100) | 0.255 | |

* Results are reported as numbers [/total number] (percentages), median (interquartile range), or mean (± standard deviation) as appropriate. P-values by Mann-Whitney U test and Pearson Chi-Squared test. COPD, chronic obstructive pulmonary disease; TIA, transient ischemic attack; ROSC, return of spontaneous circulation. To compare to the whole population included in the Target Temperature Management after out-of-hospital cardiac arrest (TTM) trial, please see Table 1 of the TTM-trial.

‡ Scores on the Glasgow Coma Scale range from 3 to 15, with lower scores indicating reduced level of consciousness.

§ Circulatory shock was defined as a systolic blood pressure of less than 90 mm Hg for more than 30 minutes or end-organ hypoperfusion (cool extremities, urine output <30 ml per hour, and a heart rate of <60 beats per minute).

¶ Missing values. ‘Glasgow Coma Scale score’ was missing data for 10 patients; ‘Serum pH’ and ‘Serum lactate’ were missing data for 8 patients.

**Table S3a.** Full list of enriched biological processes for the elevated proteins according to neurologic outcome.

| **Biological processes for the up-regulated proteins** | | | | |
| --- | --- | --- | --- | --- |
| **Group ID** | **Term** | **Description** | **p-value (log10) *** | **Proteins included** |
| 1: Summary | R-HSA-114608 | Platelet degranulation | -10,38012751 | SERPINA3,CFD,FGA,FGB,FGG,ITIH3,  EFEMP1,IGFBP2,IGFBP4,C7,B2M,UBB,  CST3, AGT, CCL14, LCN2 |
| 1 | R-HSA-76005 | Response to elevated platelet cytosolic Ca2+ | -10,2797165 | SERPINA3,CFD,FGA,FGB,FGG,ITIH3 |
| 1 | CORUM:6417 | Fibrinogen complex | -9,914112898 | FGA,FGB,FGG |
| 1 | M3008 | NABA ECM GLYCOPROTEINS | -9,280736976 | EFEMP1,FGA,FGB,FGG,IGFBP2,IGFBP4 |
| 1 | hsa04610 | Complement and coagulation cascades | -9,175877432 | C7,CFD,FGA,FGB,FGG |
| 1 | R-HSA-1236974 | ER-Phagosome pathway | -9,049479861 | B2M,FGA,FGB,FGG,UBB |
| 1 | WP2806 | Complement system | -8,839173334 | C7,CFD,FGA,FGB,FGG |
| 1 | R-HSA-1236975 | Antigen processing-Cross presentation | -8,709612168 | B2M,FGA,FGB,FGG,UBB |
| 1 | R-HSA-76002 | Platelet activation, signaling and aggregation | -8,514231874 | SERPINA3,CFD,FGA,FGB,FGG,ITIH3 |
| 1 | M5884 | NABA CORE MATRISOME | -8,398362467 | EFEMP1,FGA,FGB,FGG,IGFBP2,IGFBP4 |
| 1 | WP4927 | COVID-19, thrombosis and anticoagulation | -8,370605644 | FGA,FGB,FGG |
| 1 | R-HSA-381426 | Regulation of Insulin-like Growth Factor (IGF) transport and uptake by Insulin-like Growth Factor Binding Proteins (IGFBPs) | -8,326853574 | CST3,FGA,FGG,IGFBP2,IGFBP4 |
| 1 | GO:0031639 | plasminogen activation | -7,835913042 | FGA,FGB,FGG |
| 1 | GO:0019229 | regulation of vasoconstriction | -7,48554684 | AGT,FGA,FGB,FGG |
| 1 | GO:0072378 | blood coagulation, fibrin clot formation | -7,354553714 | FGA,FGB,FGG |
| 1 | GO:0034116 | positive regulation of heterotypic cell-cell adhesion | -7,257783905 | FGA,FGB,FGG |
| 1 | R-HSA-354194 | GRB2:SOS provides linkage to MAPK signaling for Integrins | -7,257783905 | FGA,FGB,FGG |
| 1 | R-HSA-372708 | p130Cas linkage to MAPK signaling for integrins | -7,257783905 | FGA,FGB,FGG |
| 1 | WP176 | Folate metabolism | -7,253020674 | SERPINA3,FGA,FGB,FGG |
| 1 | GO:0072376 | protein activation cascade | -7,167747479 | FGA,FGB,FGG |
| 1 | WP5115 | Network map of SARS-CoV-2 signaling pathway | -7,086756367 | AGT,FGA,FGB,FGG,ITIH3 |
| 1 | R-HSA-5602498 | MyD88 deficiency (TLR2/4) | -7,083566799 | FGA,FGB,FGG |
| 1 | GO:0042730 | fibrinolysis | -7,004525759 | FGA,FGB,FGG |
| 1 | R-HSA-5603041 | IRAK4 deficiency (TLR2/4) | -7,004525759 | FGA,FGB,FGG |
| 1 | hsa05171 | Coronavirus disease - COVID-19 | -6,981789818 | C7,CFD,FGA,FGB,FGG |
| 1 | WP15 | Selenium micronutrient network | -6,846196441 | SERPINA3,FGA,FGB,FGG |
| 1 | R-HSA-5686938 | Regulation of TLR by endogenous ligand | -6,792784899 | FGA,FGB,FGG |
| 1 | R-HSA-140875 | Common Pathway of Fibrin Clot Formation | -6,729256028 | FGA,FGB,FGG |
| 1 | R-HSA-166058 | MyD88:MAL(TIRAP) cascade initiated on plasma membrane | -6,68271956 | FGA,FGB,FGG,UBB |
| 1 | R-HSA-168188 | Toll Like Receptor TLR6:TLR2 Cascade | -6,68271956 | FGA,FGB,FGG,UBB |
| 1 | WP272 | Blood clotting cascade | -6,668698398 | FGA,FGB,FGG |
| 1 | R-HSA-168179 | Toll Like Receptor TLR1:TLR2 Cascade | -6,631524805 | FGA,FGB,FGG,UBB |
| 1 | R-HSA-181438 | Toll Like Receptor 2 (TLR2) Cascade | -6,631524805 | FGA,FGB,FGG,UBB |
| 1 | R-HSA-8957275 | Post-translational protein phosphorylation | -6,565567193 | CST3,FGA,FGG,IGFBP4 |
| 1 | GO:0034114 | regulation of heterotypic cell-cell adhesion | -6,555469544 | FGA,FGB,FGG |
| 1 | GO:0022409 | positive regulation of cell-cell adhesion | -6,493741067 | B2M,FGA,FGB,FGG,IGFBP2 |
| 1 | R-HSA-354192 | Integrin signaling | -6,451351934 | FGA,FGB,FGG |
| 1 | M169 | PID INTEGRIN2 PATHWAY | -6,354989688 | FGA,FGB,FGG |
| 1 | M257 | PID EPHRINB REV PATHWAY | -6,309372412 | FGA,FGB,FGG |
| 1 | R-HSA-109582 | Hemostasis | -6,305566002 | SERPINA3,CFD,FGA,FGB,FGG,ITIH3 |
| 1 | GO:1902042 | negative regulation of extrinsic apoptotic signaling pathway via death domain receptors | -6,265308964 | FGA,FGB,FGG |
| 1 | R-HSA-5260271 | Diseases of Immune System | -6,265308964 | FGA,FGB,FGG |
| 1 | R-HSA-5602358 | Diseases associated with the TLR signaling cascade | -6,265308964 | FGA,FGB,FGG |
| 1 | GO:0045907 | positive regulation of vasoconstriction | -6,181444731 | FGA,FGB,FGG |
| 1 | R-HSA-166016 | Toll Like Receptor 4 (TLR4) Cascade | -6,176739158 | FGA,FGB,FGG,UBB |
| 1 | GO:2000352 | negative regulation of endothelial cell apoptotic process | -6,141467725 | FGA,FGB,FGG |
| 1 | GO:0035296 | regulation of tube diameter | -6,101192102 | AGT,FGA,FGB,FGG |
| 1 | GO:0097746 | blood vessel diameter maintenance | -6,101192102 | AGT,FGA,FGB,FGG |
| 1 | GO:0010038 | response to metal ion | -6,092053033 | B2M,FGA,FGB,FGG,IGFBP2 |
| 1 | GO:0035150 | regulation of tube size | -6,088921157 | AGT,FGA,FGB,FGG |
| 1 | R-HSA-6802948 | Signaling by high-kinase activity BRAF mutants | -6,065041534 | FGA,FGB,FGG |
| 1 | GO:2001233 | regulation of apoptotic signaling pathway | -5,99683804 | AGT,FGA,FGB,FGG,UBB |
| 1 | GO:2001236 | regulation of extrinsic apoptotic signaling pathway | -5,959489802 | AGT,FGA,FGB,FGG |
| 1 | R-HSA-140877 | Formation of Fibrin Clot (Clotting Cascade) | -5,958261728 | FGA,FGB,FGG |
| 1 | R-HSA-76009 | Platelet Aggregation (Plug Formation) | -5,958261728 | FGA,FGB,FGG |
| 1 | R-HSA-983169 | Class I MHC mediated antigen processing & presentation | -5,939472829 | B2M,FGA,FGB,FGG,UBB |
| 1 | R-HSA-5674135 | MAP2K and MAPK activation | -5,924543682 | FGA,FGB,FGG |
| 1 | R-HSA-168898 | Toll-like Receptor Cascades | -5,914769717 | FGA,FGB,FGG,UBB |
| 1 | GO:1900026 | positive regulation of substrate adhesion-dependent cell spreading | -5,859639183 | FGA,FGB,FGG |
| 1 | M174 | PID UPA UPAR PATHWAY | -5,859639183 | FGA,FGB,FGG |
| 1 | GO:0070527 | platelet aggregation | -5,828370942 | FGA,FGB,FGG |
| 1 | M53 | PID INTEGRIN3 PATHWAY | -5,828370942 | FGA,FGB,FGG |
| 1 | R-HSA-9656223 | Signaling by RAF1 mutants | -5,828370942 | FGA,FGB,FGG |
| 1 | WP4136 | Fibrin complement receptor 3 signaling pathway | -5,797842346 | FGA,FGB,FGG |
| 1 | GO:0030195 | negative regulation of blood coagulation | -5,768019348 | FGA,FGB,FGG |
| 1 | GO:1900047 | negative regulation of hemostasis | -5,738870197 | FGA,FGB,FGG |
| 1 | R-HSA-6802946 | Signaling by moderate kinase activity BRAF mutants | -5,710365241 | FGA,FGB,FGG |
| 1 | R-HSA-6802949 | Signaling by RAS mutants | -5,710365241 | FGA,FGB,FGG |
| 1 | R-HSA-6802955 | Paradoxical activation of RAF signaling by kinase inactive BRAF | -5,710365241 | FGA,FGB,FGG |
| 1 | R-HSA-9649948 | Signaling downstream of RAS mutants | -5,710365241 | FGA,FGB,FGG |
| 1 | GO:0043408 | regulation of MAPK cascade | -5,705010998 | AGT,FGA,FGB,FGG,IGFBP4,CCL14 |
| 1 | GO:0050819 | negative regulation of coagulation | -5,655178723 | FGA,FGB,FGG |
| 1 | GO:0031638 | zymogen activation | -5,628446805 | FGA,FGB,FGG |
| 1 | GO:1902041 | regulation of extrinsic apoptotic signaling pathway via death domain receptors | -5,628446805 | FGA,FGB,FGG |
| 1 | GO:0045785 | positive regulation of cell adhesion | -5,581877814 | B2M,FGA,FGB,FGG,IGFBP2 |
| 1 | GO:1904036 | negative regulation of epithelial cell apoptotic process | -5,576591061 | FGA,FGB,FGG |
| 1 | GO:0022407 | regulation of cell-cell adhesion | -5,534842626 | B2M,FGA,FGB,FGG,IGFBP2 |
| 1 | GO:0045087 | innate immune response | -5,435140185 | C7,CFD,FGA,FGB,LCN2,CCL14 |
| 1 | GO:0034109 | homotypic cell-cell adhesion | -5,432485099 | FGA,FGB,FGG |
| 1 | GO:1900024 | regulation of substrate adhesion-dependent cell spreading | -5,432485099 | FGA,FGB,FGG |
| 1 | GO:2000351 | regulation of endothelial cell apoptotic process | -5,409961348 | FGA,FGB,FGG |
| 1 | WP558 | Complement and coagulation cascades | -5,409961348 | C7,CFD,FGB |
| 1 | GO:0010035 | response to inorganic substance | -5,285599426 | B2M,FGA,FGB,FGG,IGFBP2 |
| 1 | GO:0030193 | regulation of blood coagulation | -5,2624606 | FGA,FGB,FGG |
| 1 | M18 | PID INTEGRIN1 PATHWAY | -5,2624606 | FGA,FGB,FGG |
| 1 | GO:0046883 | regulation of hormone secretion | -5,248779018 | AGT,FGA,FGB,FGG |
| 1 | R-HSA-6802952 | Signaling by BRAF and RAF1 fusions | -5,242706011 | FGA,FGB,FGG |
| 1 | GO:1900046 | regulation of hemostasis | -5,223250695 | FGA,FGB,FGG |
| 1 | GO:0050818 | regulation of coagulation | -5,166593591 | FGA,FGB,FGG |
| 1 | GO:0061045 | negative regulation of wound healing | -5,130165851 | FGA,FGB,FGG |
| 1 | GO:0070374 | positive regulation of ERK1 and ERK2 cascade | -5,111902535 | FGA,FGB,FGG,CCL14 |
| 1 | GO:0043410 | positive regulation of MAPK cascade | -5,101598492 | FGA,FGB,FGG,IGFBP4,CCL14 |
| 1 | GO:1903522 | regulation of blood circulation | -5,072617582 | AGT,FGA,FGB,FGG |
| 1 | GO:0010770 | positive regulation of cell morphogenesis involved in differentiation | -5,026708612 | FGA,FGB,FGG |
| 1 | GO:0003018 | vascular process in circulatory system | -5,026450873 | AGT,FGA,FGB,FGG |
| 1 | GO:0045921 | positive regulation of exocytosis | -4,962105064 | FGA,FGB,FGG |
| 1 | GO:1903532 | positive regulation of secretion by cell | -4,956394528 | AGT,FGA,FGB,FGG |
| 1 | R-HSA-6802957 | Oncogenic MAPK signaling | -4,946451048 | FGA,FGB,FGG |
| 1 | R-HSA-216083 | Integrin cell surface interactions | -4,930986226 | FGA,FGB,FGG |
| 1 | R-HSA-5673001 | RAF/MAP kinase cascade | -4,919390595 | FGA,FGB,FGG,UBB |
| 1 | R-HSA-5684996 | MAPK1/MAPK3 signaling | -4,883193257 | FGA,FGB,FGG,UBB |
| 1 | GO:1903035 | negative regulation of response to wounding | -4,870931611 | FGA,FGB,FGG |
| 1 | GO:0006959 | humoral immune response | -4,859493094 | C7,CFD,FGA,FGB |
| 1 | GO:0090277 | positive regulation of peptide hormone secretion | -4,841930144 | FGA,FGB,FGG |
| 1 | GO:0002793 | positive regulation of peptide secretion | -4,813572397 | FGA,FGB,FGG |
| 1 | GO:0051258 | protein polymerization | -4,813572397 | FGA,FGB,FGG |
| 1 | GO:0051047 | positive regulation of secretion | -4,801684279 | AGT,FGA,FGB,FGG |
| 1 | GO:1904035 | regulation of epithelial cell apoptotic process | -4,799626193 | FGA,FGB,FGG |
| 1 | R-HSA-1474244 | Extracellular matrix organization | -4,796012855 | EFEMP1,FGA,FGB,FGG |
| 1 | GO:0010769 | regulation of cell morphogenesis involved in differentiation | -4,772182647 | FGA,FGB,FGG |
| 1 | GO:0030168 | platelet activation | -4,772182647 | FGA,FGB,FGG |
| 1 | GO:2001237 | negative regulation of extrinsic apoptotic signaling pathway | -4,758679026 | FGA,FGB,FGG |
| 1 | R-HSA-5683057 | MAPK family signaling cascades | -4,665428806 | FGA,FGB,FGG,UBB |
| 1 | GO:0009617 | response to bacterium | -4,557422275 | B2M,CFD,FGA,FGB,LCN2 |
| 1 | GO:0070372 | regulation of ERK1 and ERK2 cascade | -4,521755776 | FGA,FGB,FGG,CCL14 |
| 1 | GO:0030155 | regulation of cell adhesion | -4,462315314 | B2M,FGA,FGB,FGG,IGFBP2 |
| 1 | GO:0010811 | positive regulation of cell-substrate adhesion | -4,460625847 | FGA,FGB,FGG |
| 1 | GO:0046887 | positive regulation of hormone secretion | -4,460625847 | FGA,FGB,FGG |
| 1 | R-HSA-1280218 | Adaptive Immune System | -4,454139342 | B2M,FGA,FGB,FGG,UBB |
| 1 | hsa04611 | Platelet activation | -4,43954622 | FGA,FGB,FGG |
| 1 | GO:0050714 | positive regulation of protein secretion | -4,398409725 | FGA,FGB,FGG |
| 1 | GO:0007160 | cell-matrix adhesion | -4,358566506 | FGA,FGB,FGG |
| 1 | GO:0061041 | regulation of wound healing | -4,3487985 | FGA,FGB,FGG |
| 1 | GO:0008015 | blood circulation | -4,313614711 | AGT,FGA,FGB,FGG |
| 1 | GO:0051592 | response to calcium ion | -4,246051646 | FGA,FGB,FGG |
| 1 | R-HSA-5663202 | Diseases of signal transduction by growth factor receptors and second messengers | -4,179985682 | FGA,FGB,FGG,UBB |
| 1 | GO:1903034 | regulation of response to wounding | -4,062689174 | FGA,FGB,FGG |
| 1 | GO:0007596 | blood coagulation | -4,01699341 | FGA,FGB,FGG |
| 1 | GO:0050817 | coagulation | -4,002123707 | FGA,FGB,FGG |
| 1 | GO:0090066 | regulation of anatomical structure size | -3,982652798 | AGT,FGA,FGB,FGG |
| 1 | GO:0007599 | hemostasis | -3,980144452 | FGA,FGB,FGG |
| 1 | GO:0090276 | regulation of peptide hormone secretion | -3,980144452 | FGA,FGB,FGG |
| 1 | GO:0003013 | circulatory system process | -3,965548511 | AGT,FGA,FGB,FGG |
| 1 | GO:0002791 | regulation of peptide secretion | -3,958543832 | FGA,FGB,FGG |
| 1 | GO:0010817 | regulation of hormone levels | -3,951995269 | AGT,FGA,FGB,FGG |
| 1 | GO:0016485 | protein processing | -3,944347529 | FGA,FGB,FGG |
| 1 | GO:0060284 | regulation of cell development | -3,938555728 | B2M,FGA,FGB,FGG |
| 1 | GO:0090087 | regulation of peptide transport | -3,937309259 | FGA,FGB,FGG |
| 1 | GO:0031589 | cell-substrate adhesion | -3,902699492 | FGA,FGB,FGG |
| 1 | hsa04613 | Neutrophil extracellular trap formation | -3,889119274 | FGA,FGB,FGG |
| 1 | GO:0060627 | regulation of vesicle-mediated transport | -3,844357941 | B2M,FGA,FGB,FGG |
| 1 | GO:0017157 | regulation of exocytosis | -3,797979478 | FGA,FGB,FGG |
| 1 | GO:1903530 | regulation of secretion by cell | -3,782492557 | AGT,FGA,FGB,FGG |
| 1 | GO:0010810 | regulation of cell-substrate adhesion | -3,718929416 | FGA,FGB,FGG |
| 1 | GO:0044057 | regulation of system process | -3,711302648 | AGT,FGA,FGB,FGG |
| 1 | GO:2001234 | negative regulation of apoptotic signaling pathway | -3,661367931 | FGA,FGB,FGG |
| 1 | GO:0051046 | regulation of secretion | -3,618374672 | AGT,FGA,FGB,FGG |
| 1 | GO:0002250 | adaptive immune response | -3,588578897 | B2M,C7,FGA,FGB |
| 1 | GO:0001775 | cell activation | -3,538410024 | B2M,FGA,FGB,FGG |
| 1 | GO:0051604 | protein maturation | -3,533240034 | FGA,FGB,FGG |
| 1 | GO:0050708 | regulation of protein secretion | -3,528179196 | FGA,FGB,FGG |
| 1 | GO:0051222 | positive regulation of protein transport | -3,336805106 | FGA,FGB,FGG |
| 1 | GO:0010720 | positive regulation of cell development | -3,319639916 | FGA,FGB,FGG |
| 1 | GO:0022604 | regulation of cell morphogenesis | -3,286017119 | FGA,FGB,FGG |
| 1 | GO:1904951 | positive regulation of establishment of protein localization | -3,269547151 | FGA,FGB,FGG |
| 1 | GO:0042060 | wound healing | -3,20193545 | FGA,FGB,FGG |
| 1 | GO:0042742 | defense response to bacterium | -3,12330525 | FGA,FGB,LCN2 |
| 1 | GO:0050878 | regulation of body fluid levels | -3,056322482 | FGA,FGB,FGG |
| 1 | GO:0032102 | negative regulation of response to external stimulus | -2,863538067 | FGA,FGB,FGG |
| 1 | GO:0009611 | response to wounding | -2,854816316 | FGA,FGB,FGG |
| 1 | WP3888 | VEGFA-VEGFR2 signaling pathway | -2,829029876 | FGA,FGB,FGG |
| 1 | GO:1903829 | positive regulation of protein localization | -2,809355329 | FGA,FGB,FGG |
| 1 | GO:0051223 | regulation of protein transport | -2,680137366 | FGA,FGB,FGG |
| 1 | GO:0070201 | regulation of establishment of protein localization | -2,619328291 | FGA,FGB,FGG |
| 1 | GO:0098609 | cell-cell adhesion | -2,588921913 | FGA,FGB,FGG |
| 2: Summary | R-HSA-977225 | Amyloid fiber formation | -6,517718726 | B2M,CST3,FGA,UBB,SERPINA3,LCN2 |
| 2 | GO:0048871 | multicellular organismal homeostasis | -3,080569192 | SERPINA3,B2M,UBB |
| 2 | R-HSA-1280215 | Cytokine Signaling in Immune system | -2,23114061 | B2M,LCN2,UBB |
| 3: Summary | M5885 | NABA MATRISOME ASSOCIATED | -5,478054478 | SERPINA3,AGT,CST3,ITIH3,CCL14,  SFTPB,UBB |
| 3 | M3468 | NABA ECM REGULATORS | -5,197553458 | SERPINA3,AGT,CST3,ITIH3 |
| 3 | GO:0010951 | negative regulation of endopeptidase activity | -5,106394551 | SERPINA3,AGT,CST3,ITIH3 |
| 3 | GO:0010466 | negative regulation of peptidase activity | -5,032968308 | SERPINA3,AGT,CST3,ITIH3 |
| 3 | GO:0045861 | negative regulation of proteolysis | -4,539580718 | SERPINA3,AGT,CST3,ITIH3 |
| 3 | GO:0030162 | regulation of proteolysis | -4,509296806 | SERPINA3,AGT,CST3,ITIH3,UBB |
| 3 | GO:0051346 | negative regulation of hydrolase activity | -4,44084658 | SERPINA3,AGT,CST3,ITIH3 |
| 3 | GO:0052548 | regulation of endopeptidase activity | -4,207426211 | SERPINA3,AGT,CST3,ITIH3 |
| 3 | GO:0052547 | regulation of peptidase activity | -4,092967409 | SERPINA3,AGT,CST3,ITIH3 |
| 3 | GO:0043086 | negative regulation of catalytic activity | -3,190673394 | SERPINA3,AGT,CST3,ITIH3 |
| 4: Summary | R-HSA-6798695 | Neutrophil degranulation | -5,426173723 | SERPINA3,B2M,CST3,CFD,LCN2,C7 |
| 4 | GO:0002252 | immune effector process | -2,860623681 | B2M,C7,CFD |
| 4 | GO:0050778 | positive regulation of immune response | -2,530407354 | B2M,C7,CFD |
| 5: Summary | GO:0007568 | aging | -3,454660106 | AGT,B2M,IGFBP2,FGB,IGFBP4 |
| 5 | GO:0032870 | cellular response to hormone stimulus | -2,744287862 | AGT,FGB,IGFBP2 |
| 5 | GO:0040008 | regulation of growth | -2,327847738 | AGT,IGFBP2,IGFBP4 |
| 5 | GO:0009725 | response to hormone | -2,186576407 | AGT,FGB,IGFBP2 |
| 6: Summary | GO:0055065 | metal ion homeostasis | -3,374367019 | AGT,B2M,LCN2,CCL14 |
| 6 | GO:0055080 | cation homeostasis | -3,189451216 | AGT,B2M,LCN2,CCL14 |
| 6 | GO:0098771 | inorganic ion homeostasis | -3,166096646 | AGT,B2M,LCN2,CCL14 |
| 6 | GO:0050801 | ion homeostasis | -3,050146933 | AGT,B2M,LCN2,CCL14 |
| 7: Summary | GO:0050865 | regulation of cell activation | -2,289929035 | B2M,FGG,IGFBP2 |
| 7 | GO:0050865 | regulation of cell activation | -2,289929035 | B2M,FGG,IGFBP2 |

*p-value (log10), i.e., -2 represents 0.01, the more negative the better.

**Abbreviations:** AGT – Angiotensinogen; B2M - Beta-2-microglobulin; C7 - Complement component C7; CCL14 - C-C motif chemokine 14; CFD - Complement factor D; CST3 - Cystatin-C; EFEMP1 - EGF-containing fibulin-like extracellular matrix protein 1; FGA - Fibrinogen alpha chain; FGB - Fibrinogen beta chain; FGG - Fibrinogen gamma chain; IGFBP2 - Insulin-like growth factor-binding protein 2; IGFBP4 - Insulin-like growth factor-binding protein 4; IGHV3-23 - Immunoglobulin heavy variable 3-23; ITIH3 - Inter-alpha-trypsin inhibitor heavy chain H3; LCN2 - Neutrophil gelatinase-associated lipocalin; RNASE1 - Ribonuclease pancreatic;SERPINA3 - Alpha-1-antichymotrypsin; SFTPB - Pulmonary surfactant-associated protein B; UBB / UBC - Polyubiquitin-B / Polyubiquitin-C.

**Table S3b.** Full list of enriched biological processes for the reduced proteins according to neurologic outcome.

| **Biological processes for the down-regulated proteins** | | | | |
| --- | --- | --- | --- | --- |
| **Group ID** | **Term** | **Description** | **p-value (log10) *** | **Proteins included** |
| 8: Summary | GO:0030162 | regulation of proteolysis | -9,147313361 | C3,GPLD1,GSN,ITIH1,ITIH2,KLKB1,  SERPINA4,FETUB,PLG |
| 8 | GO:0052548 | regulation of endopeptidase activity | -7,467094688 | C3,GSN,ITIH1,ITIH2,SERPINA4,  FETUB |
| 8 | GO:0052547 | regulation of peptidase activity | -7,292111068 | C3,GSN,ITIH1,ITIH2,SERPINA4,  FETUB |
| 8 | GO:0010951 | negative regulation of endopeptidase activity | -6,971717179 | C3,ITIH1,ITIH2,SERPINA4,FETUB |
| 8 | GO:0010466 | negative regulation of peptidase activity | -6,879169198 | C3,ITIH1,ITIH2,SERPINA4,FETUB |
| 8 | GO:0045861 | negative regulation of proteolysis | -6,256735618 | C3,ITIH1,ITIH2,SERPINA4,FETUB |
| 8 | GO:0051346 | negative regulation of hydrolase activity | -6,132032789 | C3,ITIH1,ITIH2,SERPINA4,FETUB |
| 8 | M3468 | NABA ECM REGULATORS | -5,31979937 | ITIH1,ITIH2,SERPINA4,PLG |
| 8 | GO:0043086 | negative regulation of catalytic activity | -4,545885794 | C3,ITIH1,ITIH2,SERPINA4,FETUB |
| 8 | M5885 | NABA MATRISOME ASSOCIATED | -3,381417178 | ITIH1,ITIH2,SERPINA4,PLG |
| 9: Summary | WP558 | Complement and coagulation cascades | -5,813311401 | C3,KLKB1,PLG,PROZ,TNXB,  SERPINA4 |
| 9 | hsa04610 | Complement and coagulation cascades | -5,333215363 | C3,KLKB1,PLG |
| 9 | WP2806 | Complement system | -5,133718809 | C3,KLKB1,PLG |
| 9 | GO:0007596 | blood coagulation | -4,415474049 | KLKB1,PLG,PROZ |
| 9 | GO:0050817 | coagulation | -4,400518216 | KLKB1,PLG,PROZ |
| 9 | GO:0007599 | hemostasis | -4,378409769 | KLKB1,PLG,PROZ |
| 9 | R-HSA-1474244 | Extracellular matrix organization | -3,695641937 | KLKB1,PLG,TNXB |
| 9 | GO:0042060 | wound healing | -3,593789824 | KLKB1,PLG,PROZ |
| 9 | GO:0050878 | regulation of body fluid levels | -3,446457741 | KLKB1,PLG,PROZ |
| 9 | GO:0009611 | response to wounding | -3,24220279 | KLKB1,PLG,PROZ |
| 9 | R-HSA-109582 | Hemostasis | -2,781044484 | KLKB1,SERPINA4,PLG |
| 10: Summary | GO:0046486 | glycerolipid metabolic process | -3,478109469 | GPLD1,TNXB,PLA2G7 |
| 10 | GO:0046486 | glycerolipid metabolic process | -3,478109469 | GPLD1,TNXB,PLA2G7 |
| 11: Summary | GO:0050727 | regulation of inflammatory response | -3,352887358 | C3,KLKB1,PLA2G7,PLG |
| 11 | GO:0032103 | positive regulation of response to external stimulus | -3,207429116 | C3,PLG,PLA2G7 |
| 11 | GO:0031347 | regulation of defense response | -2,72814555 | C3,KLKB1,PLA2G7 |

*p-value (log10), i.e., -2 represents 0.01, the more negative the better.

**Abbreviations:** AFM – Afamin; C3 - Complement C3; FETUB - Fetuin-B; GPLD1 - Phosphatidylinositol-glycan-specific phospholipase D; GSN – Gelsolin; ITIH1 - Inter-alpha-trypsin inhibitor heavy chain H1; ITIH2 - Inter-alpha-trypsin inhibitor heavy chain H2; KLKB1 - Plasma kallikrein; LPA – Apolipoprotein (a); PEPD - Xaa-Pro dipeptidase; PLA2G7 - Platelet-activating factor acetylhydrolase; PLG – Plasminogen; PROZ - Vitamin K-dependent protein Z; SERPINA4 – Kallistatin; TNXB - Tenascin-X.

**
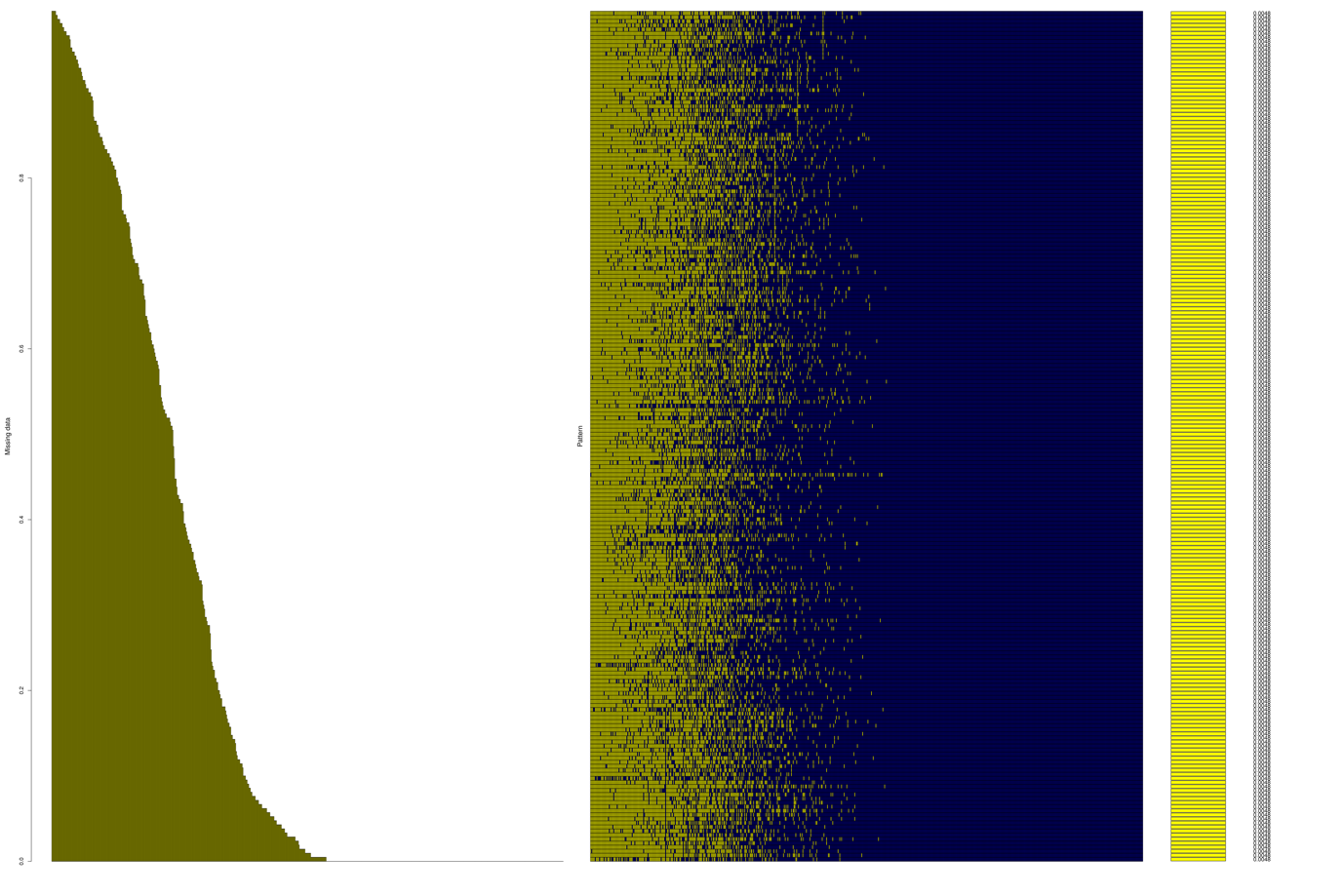
**

**Figure S1.** QC plots for the missing protein values in the proteomics data. Yellow color indicates proteins missing across samples in the heatmap (right). Barplot (left) shows frequencies of the missing protein values, indicating that the proteins are not present in consistently detectable amount.

**References**

1. Lichenstein HS, Lyons DE, Wurfel MM, Johnson DA, McGinley MD, Leidli JC, et al. Afamin is a new member of the albumin, alpha-fetoprotein, and vitamin D-binding protein gene family. J Biol Chem. 1994;269(27):18149-54.

2. Weir MR, Dzau VJ. The renin-angiotensin-aldosterone system: a specific target for hypertension management. Am J Hypertens. 1999;12(12 Pt 3):205s-13s.

3. Yu B, Thompson GD, Yip P, Howell PL, Davidson AR. Mechanisms for intragenic complementation at the human argininosuccinate lyase locus. Biochemistry. 2001;40(51):15581-90.

4. Güssow D, Rein R, Ginjaar I, Hochstenbach F, Seemann G, Kottman A, et al. The human beta 2-microglobulin gene. Primary structure and definition of the transcriptional unit. J Immunol. 1987;139(9):3132-8.

5. de Bruijn MH, Fey GH. Human complement component C3: cDNA coding sequence and derived primary structure. Proc Natl Acad Sci U S A. 1985;82(3):708-12.

6. DiScipio RG, Chakravarti DN, Muller-Eberhard HJ, Fey GH. The structure of human complement component C7 and the C5b-7 complex. J Biol Chem. 1988;263(1):549-60.

7. Detheux M, Ständker L, Vakili J, Münch J, Forssmann U, Adermann K, et al. Natural proteolytic processing of hemofiltrate CC chemokine 1 generates a potent CC chemokine receptor (CCR)1 and CCR5 agonist with anti-HIV properties. J Exp Med. 2000;192(10):1501-8.

8. Fearon DT, Austen KF. Initiation of C3 cleavage in the alternative complement pathway. J Immunol. 1975;115(5):1357-61.

9. Mahata SK, Corti A. Chromogranin A and its fragments in cardiovascular, immunometabolic, and cancer regulation. Ann N Y Acad Sci. 2019;1455(1):34-58.

10. Lee CG, Hartl D, Lee GR, Koller B, Matsuura H, Da Silva CA, et al. Role of breast regression protein 39 (BRP-39)/chitinase 3-like-1 in Th2 and IL-13-induced tissue responses and apoptosis. J Exp Med. 2009;206(5):1149-66.

11. Abrahamson M, Barrett AJ, Salvesen G, Grubb A. Isolation of six cysteine proteinase inhibitors from human urine. Their physicochemical and enzyme kinetic properties and concentrations in biological fluids. J Biol Chem. 1986;261(24):11282-9.

12. Camaj P, Seeliger H, Ischenko I, Krebs S, Blum H, De Toni EN, et al. EFEMP1 binds the EGF receptor and activates MAPK and Akt pathways in pancreatic carcinoma cells. Biol Chem. 2009;390(12):1293-302.

13. Gaudet P, Livstone MS, Lewis SE, Thomas PD. Phylogenetic-based propagation of functional annotations within the Gene Ontology consortium. Brief Bioinform. 2011;12(5):449-62.

14. Doolittle RF. Fibrinogen and fibrin. Annu Rev Biochem. 1984;53:195-229.

15. Davitz MA, Hereld D, Shak S, Krakow J, Englund PT, Nussenzweig V. A glycan-phosphatidylinositol-specific phospholipase D in human serum. Science. 1987;238(4823):81-4.

16. Kwiatkowski DJ, Stossel TP, Orkin SH, Mole JE, Colten HR, Yin HL. Plasma and cytoplasmic gelsolins are encoded by a single gene and contain a duplicated actin-binding domain. Nature. 1986;323(6087):455-8.

17. Zhou J, Li W, Kamei H, Duan C. Duplication of the IGFBP-2 gene in teleost fish: protein structure and functionality conservation and gene expression divergence. PLoS One. 2008;3(12):e3926.

18. LaTour D, Mohan S, Linkhart TA, Baylink DJ, Strong DD. Inhibitory insulin-like growth factor-binding protein: cloning, complete sequence, and physiological regulation. Mol Endocrinol. 1990;4(12):1806-14.

19. Lefranc MP. Immunoglobulin and T Cell Receptor Genes: IMGT(®) and the Birth and Rise of Immunoinformatics. Front Immunol. 2014;5:22.

20. Diarra-Mehrpour M, Bourguignon J, Bost F, Sesboüé R, Muschio F, Sarafan N, et al. Human inter-alpha-trypsin inhibitor: full-length cDNA sequence of the heavy chain H1. Biochim Biophys Acta. 1992;1132(1):114-8.

21. Miyata T, Kawabata S, Iwanaga S, Takahashi I, Alving B, Saito H. Coagulation factor XII (Hageman factor) Washington D.C.: inactive factor XIIa results from Cys-571----Ser substitution. Proc Natl Acad Sci U S A. 1989;86(21):8319-22.

22. Beaubien G, Rosinski-Chupin I, Mattei MG, Mbikay M, Chrétien M, Seidah NG. Gene structure and chromosomal localization of plasma kallikrein. Biochemistry. 1991;30(6):1628-35.

23. Yang J, Goetz D, Li JY, Wang W, Mori K, Setlik D, et al. An iron delivery pathway mediated by a lipocalin. Mol Cell. 2002;10(5):1045-56.

24. Shields-Cutler RR, Crowley JR, Miller CD, Stapleton AE, Cui W, Henderson JP. Human Metabolome-derived Cofactors Are Required for the Antibacterial Activity of Siderocalin in Urine. J Biol Chem. 2016;291(50):25901-10.

25. Bao G, Clifton M, Hoette TM, Mori K, Deng SX, Qiu A, et al. Iron traffics in circulation bound to a siderocalin (Ngal)-catechol complex. Nat Chem Biol. 2010;6(8):602-9.

26. Salonen EM, Jauhiainen M, Zardi L, Vaheri A, Ehnholm C. Lipoprotein(a) binds to fibronectin and has serine proteinase activity capable of cleaving it. Embo j. 1989;8(13):4035-40.

27. Boyd RS, Adam PJ, Patel S, Loader JA, Berry J, Redpath NT, et al. Proteomic analysis of the cell-surface membrane in chronic lymphocytic leukemia: identification of two novel proteins, BCNP1 and MIG2B. Leukemia. 2003;17(8):1605-12.

28. Tanoue A, Endo F, Matsuda I. Structural organization of the gene for human prolidase (peptidase D) and demonstration of a partial gene deletion in a patient with prolidase deficiency. J Biol Chem. 1990;265(19):11306-11.

29. Tjoelker LW, Wilder C, Eberhardt C, Stafforini DM, Dietsch G, Schimpf B, et al. Anti-inflammatory properties of a platelet-activating factor acetylhydrolase. Nature. 1995;374(6522):549-53.

30. Rossignol P, Ho-Tin-Noé B, Vranckx R, Bouton MC, Meilhac O, Lijnen HR, et al. Protease nexin-1 inhibits plasminogen activation-induced apoptosis of adherent cells. J Biol Chem. 2004;279(11):10346-56.

31. Ichinose A, Takeya H, Espling E, Iwanaga S, Kisiel W, Davie EW. Amino acid sequence of human protein Z, a vitamin K-dependent plasma glycoprotein. Biochem Biophys Res Commun. 1990;172(3):1139-44.

32. Johnson RJ, McCoy JG, Bingman CA, Phillips GN, Jr., Raines RT. Inhibition of human pancreatic ribonuclease by the human ribonuclease inhibitor protein. J Mol Biol. 2007;368(2):434-49.

33. Park GT, Lim SE, Jang SI, Morasso MI. Suprabasin, a novel epidermal differentiation marker and potential cornified envelope precursor. J Biol Chem. 2002;277(47):45195-202.

34. Chai KX, Chen LM, Chao J, Chao L. Kallistatin: a novel human serine proteinase inhibitor. Molecular cloning, tissue distribution, and expression in Escherichia coli. J Biol Chem. 1993;268(32):24498-505.

35. Whitsett JA, Glasser SW. Regulation of surfactant protein gene transcription. Biochim Biophys Acta. 1998;1408(2-3):303-11.

36. Egging D, van den Berkmortel F, Taylor G, Bristow J, Schalkwijk J. Interactions of human tenascin-X domains with dermal extracellular matrix molecules. Arch Dermatol Res. 2007;298(8):389-96.

37. Yu Y, Zheng Q, Erramilli SK, Pan M, Park S, Xie Y, et al. K29-linked ubiquitin signaling regulates proteotoxic stress response and cell cycle. Nat Chem Biol. 2021;17(8):896-905.

38. Huang F, Kirkpatrick D, Jiang X, Gygi S, Sorkin A. Differential regulation of EGF receptor internalization and degradation by multiubiquitination within the kinase domain. Mol Cell. 2006;21(6):737-48.

39. Xu ZP, Tsuji T, Riordan JF, Hu GF. The nuclear function of angiogenin in endothelial cells is related to rRNA production. Biochem Biophys Res Commun. 2002;294(2):287-92.

40. Dickson KA, Kang DK, Kwon YS, Kim JC, Leland PA, Kim BM, et al. Ribonuclease inhibitor regulates neovascularization by human angiogenin. Biochemistry. 2009;48(18):3804-6.

41. Fitzgerald J, Rich C, Zhou FH, Hansen U. Three novel collagen VI chains, alpha4(VI), alpha5(VI), and alpha6(VI). J Biol Chem. 2008;283(29):20170-80.

42. Yamamoto KK, Pousette A, Chow P, Wilson H, el Shami S, French CK. Isolation of a cDNA encoding a human serum marker for acute pancreatitis. Identification of pancreas-specific protein as pancreatic procarboxypeptidase B. J Biol Chem. 1992;267(4):2575-81.

43. Matsushita M, Endo Y, Fujita T. Cutting edge: complement-activating complex of ficolin and mannose-binding lectin-associated serine protease. J Immunol. 2000;164(5):2281-4.

44. Garlatti V, Belloy N, Martin L, Lacroix M, Matsushita M, Endo Y, et al. Structural insights into the innate immune recognition specificities of L- and H-ficolins. Embo j. 2007;26(2):623-33.

45. van Buul JD, Allingham MJ, Samson T, Meller J, Boulter E, García-Mata R, et al. RhoG regulates endothelial apical cup assembly downstream from ICAM1 engagement and is involved in leukocyte trans-endothelial migration. J Cell Biol. 2007;178(7):1279-93.

46. Hayashi T, Takahashi T, Motoya S, Ishida T, Itoh F, Adachi M, et al. MUC1 mucin core protein binds to the domain 1 of ICAM-1. Digestion. 2001;63 Suppl 1:87-92.

47. Kashyap RS, Nayak AR, Deshpande PS, Kabra D, Purohit HJ, Taori GM, et al. Inter-alpha-trypsin inhibitor heavy chain 4 is a novel marker of acute ischemic stroke. Clin Chim Acta. 2009;402(1-2):160-3.

48. Rooryck C, Diaz-Font A, Osborn DP, Chabchoub E, Hernandez-Hernandez V, Shamseldin H, et al. Mutations in lectin complement pathway genes COLEC11 and MASP1 cause 3MC syndrome. Nat Genet. 2011;43(3):197-203.

49. Dahl MR, Thiel S, Matsushita M, Fujita T, Willis AC, Christensen T, et al. MASP-3 and its association with distinct complexes of the mannan-binding lectin complement activation pathway. Immunity. 2001;15(1):127-35.

50. Nassoury N, Blasiole DA, Tebon Oler A, Benjannet S, Hamelin J, Poupon V, et al. The cellular trafficking of the secretory proprotein convertase PCSK9 and its dependence on the LDLR. Traffic. 2007;8(6):718-32.

51. Poirier S, Mayer G, Benjannet S, Bergeron E, Marcinkiewicz J, Nassoury N, et al. The proprotein convertase PCSK9 induces the degradation of low density lipoprotein receptor (LDLR) and its closest family members VLDLR and ApoER2. J Biol Chem. 2008;283(4):2363-72.
